# Solid Lipid Nanoparticles (SLNs): An Advanced Drug Delivery System Targeting Brain through BBB

**DOI:** 10.3390/pharmaceutics13081183

**Published:** 2021-07-31

**Authors:** Mantosh Kumar Satapathy, Ting-Lin Yen, Jing-Shiun Jan, Ruei-Dun Tang, Jia-Yi Wang, Rajeev Taliyan, Chih-Hao Yang

**Affiliations:** 1 Department of Pharmacology, School of Medicine, College of Medicine, Taipei Medical University, No. 250, Wu Hsing St., Taipei 110, Taiwan; mantoshbiotech@gmail.com (M.K.S.); d119096015@tmu.edu.tw (T.-L.Y.); d119101004@tmu.edu.tw (J.-S.J.); tang0803@tmu.edu.tw (R.-D.T.); 2 Department of Medical Research, Cathay General Hospital, Taipei 22174, Taiwan; 3 Graduate Institute of Medical Sciences, College of Medicine, Taipei Medical University, No. 250, Wu Hsing St., Taipei 110, Taiwan; jywang2010@tmu.edu.tw; 4 Department of Neurosurgery, Taipei Medical University Hospital, Taipei 110, Taiwan; 5 Neuroscience Research Center, Taipei Medical University, Taipei 110, Taiwan; 6 Department of Pharmacy, Neuropsychopharmacology Division, Birla Institute of Technology and Science, Pilani 333031, India; taliyanraja@gmail.com

**Keywords:** neurological disorders, BBB, nano drug delivery, SLN

## Abstract

The blood–brain barrier (BBB) plays a vital role in the protection and maintenance of homeostasis in the brain. In this way, it is an interesting target as an interface for various types of drug delivery, specifically in the context of the treatment of several neuropathological conditions where the therapeutic agents cannot cross the BBB. Drug toxicity and on-target specificity are among some of the limitations associated with current neurotherapeutics. In recent years, advances in nanodrug delivery have enabled the carrier system containing the active therapeutic drug to target the signaling pathways and pathophysiology that are closely linked to central nervous system (CNS) disorders such as Alzheimer’s disease (AD), Parkinson’s disease (PD), Huntington’s disease (HD), multiple sclerosis (MS), brain tumor, epilepsy, ischemic stroke, and neurodegeneration. At present, among the nano formulations, solid lipid nanoparticles (SLNs) have emerged as a putative drug carrier system that can deliver the active therapeutics (drug-loaded SLNs) across the BBB at the target site of the brain, offering a novel approach with controlled drug delivery, longer circulation time, target specificity, and higher efficacy, and more importantly, reducing toxicity in a biomimetic way. This paper highlights the synthesis and application of SLNs as a novel nontoxic formulation strategy to carry CNS drugs across the BBB to improve the use of therapeutics agents in treating major neurological disorders in future clinics.

## 1. Introduction

An abundance of people worldwide are affected by various chronic neurological disorders such as Alzheimer’s disease (AD), Parkinson’s disease (PD), brain tumors/cancers, Huntington’s disease (HD), neuromuscular disease, multiple sclerosis (MS), neurodegeneration, and epilepsy, resulting in tremendous morbidity and mortality [1]. Central nervous system (CNS) diseases are most commonly characterized by an imbalance in neurological function, leading to neuronal death [2,3,4]. There are multiple mechanisms associated with these neuropathologies. CNS disorders are the result of mitochondrial dysfunction, the accumulation of misfolded protein, a lack of neurotrophic factor production, endogenous antioxidant enzyme activity depletion, neurotrophin deficiency, and sometimes defects at the genetic and molecular levels. Therefore, it is very challenging to find out certain specific treatment strategy to target the CNS pathologies. The blood–brain barrier (BBB) plays a further vital role as an obstacle for the potential drugs to cross [5]. Hence, pharmacokinetic efficacy found in existing drugs is discouraging in the treatment of brain disorders. The BBB needs to be studied in detail for the development of drug and carrier systems to deliver the drug to the brain site with long-term efficacy and less possible toxicity. Less successful treatment strategies have been developed to date for the treatment of neurodegenerative diseases [6]. Simultaneously, advanced drug delivery systems such as polymer-based nano-carrier-mediated drug delivery haves been developed as a front line clinical therapeutic method which can overcome the BBB-associated hindrances. However, the limited availability and high cost of safe polymers have limited the wide-spread application of polymeric nano-formulations in clinics [7]. Solid lipid nanoparticles (SLNs) are one of the safest and cheapest carriers of the drug, enabling the treatment of neurological disorders in a nontoxic, safe, and effective way by crossing the BBB. As SLNs’ functionality and efficacy depend on their constituent, size, structure, physico-chemical properties, and the synthetic methods by which they are produced, it is necessary to shed light on the advanced production technologies used to create SLNs in the field of drug delivery. Progressively, newly formed lipid nanoparticles have overcome the shortcomings of previous SLNs [8]. The second-generation nano lipid carriers (NLCs), as modified SLNs, serve as better drug delivery carriers, overcoming limitations such as drug expulsion, and a sudden release of active drug components for brain drug delivery. Tailoring SLNs to enhance drug delivery to the brain may enable them to cross the BBB and improve the drug’s bioavailability.

Therefore, it is important to explore the properties of the BBB in detail, and to ensure that SLNs and their modifications function as an appropriate nanocarrier drug delivery system, with the potential to treat neurological disorders with less toxicity and fewer side effects.

## 2. Blood–Brain Barrier

**Figure 1:**
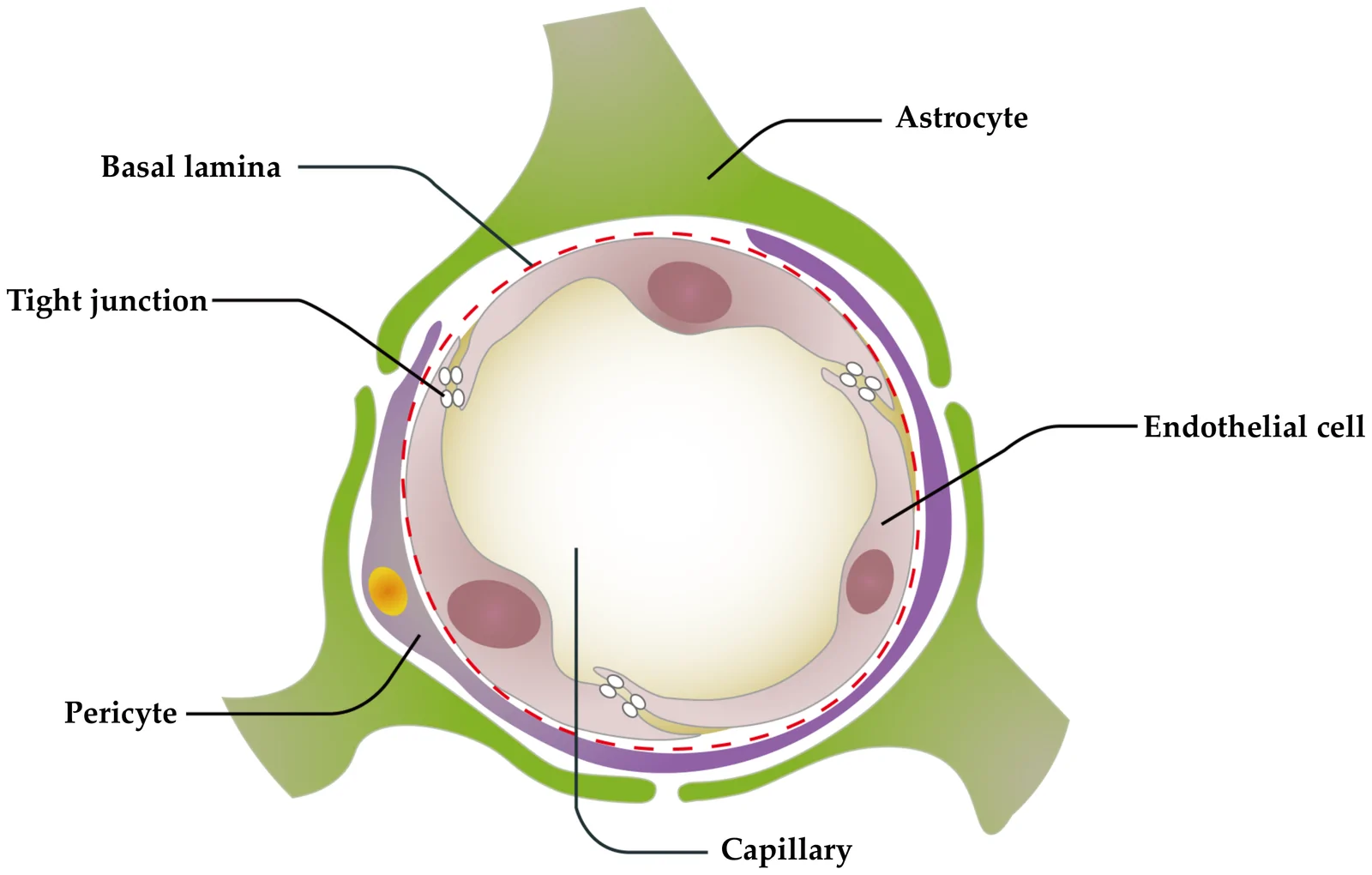
Blood brain barrier detailed anatomy showing capillary, tight junction, endothelial cell, pericyte, basal lamina, and astrocyte.

There are several anatomical and metabolic barriers present between peripheral blood circulation and the brain, such as cerebrospinal fluid (CSF), choroid plexus (CP), and the BBB. The BBB is an anatomical protective barrier to the brain which separates the brain from direct contact with the blood [9]. The BBB is a vital component of the neurovascular unit of the body, communicating CNS. At the same time, it restricts the free exchange of substances in brain cells. The main BBB components are endothelial cells, astrocytic end-feet links, basal lamina, tight junctions, and pericytes [10,11,12] (Figure 1). Most importantly, the BBB endothelial cells are quite different from peripheral endothelial cells. The BBB strictly controls the transportation of substances into the brain through both physical barriers, that is, tight junctions (composed of several transmembrane proteins, such as occludin, claudins, and junctional adhesion molecules (JAMs)), and the metabolic barrier (various enzymes). There are also many associated junctions and membrane systems, such as adherens, gap junctions, tight junctions, pericyte endothelial junctions, astrocyte junction, and the basement membrane. The essential neurovascular unit cell types that contribute to the functionality of the BBB are endothelial cell adhesion molecules, pericytes, smooth muscle cells, astrocytes, and micro glia, etc., which form the BBB, a complex barrier unit [13,14,15,16]. Transport through the BBB occurs based on endogenous-carrier- mediated transport (CMT) or a receptor-mediated transport (RMT) system [17], with the assistance of some major transporters, receptors, and channels in endothelial cells and pericytes. In this way, the BBB acts as a physical and metabolic barrier as well as a secretory and transport interface [18] to the brain and CNS. The BBB plays a major role in the regulated and specialized transport of several neurological factors from serum, allowing only useful and specific substances to cross through and protecting the brain from neurotoxins [5]. The selective transportation of nutrients or drugs across the BBB occurs through a specialized transportation across the capillary endothelial plasma membrane comprising active efflux systems, active transporters, and ectoenzymes [19]. Although many drugs are used for the treatment of neurological disorders, the specialized microvasculature of the BBB allows for the selection of a few medicines during systemic administration, which results in inadequate efficacy.

Several factors are responsible for drugs crossing the BBB, such as drug-related factors; molecular weight (below 400 Da), morphology (spherical), size (nano meter range), ionization (physiological pH), lipophilicity are the major factors [20]. The associated peripheral factors such as logP o/w value of drug (−0.5 to 6.0), enzymatic stability, plasma-protein-binding affinity, uptake of the drug into other tissues, volume of distribution, clearance rate, and rate of oxidative metabolism by cytochrome P450 effects [20,21]. These physicochemical factors, and any other pathological abnormalities that may exist, should be taken into consideration.

The regulation of BBB transport is correlated with neuronal functions such as neural degeneration and neurogenesis and contributes to the sole function of CNS. Consequently, the BBB is an interesting target to focus on in the drug design process, in which the complex pathophysiology of the human brain needs to be studied, with a greater focus on the BBB in particular. A better understanding of the BBB and a focus on developing newer small molecules, advanced drugs and carriers are the goals of the current clinical research in biomedicine targeting neurological disorders.

## 3. Brain Drug Delivery Strategies

**Figure 2:**
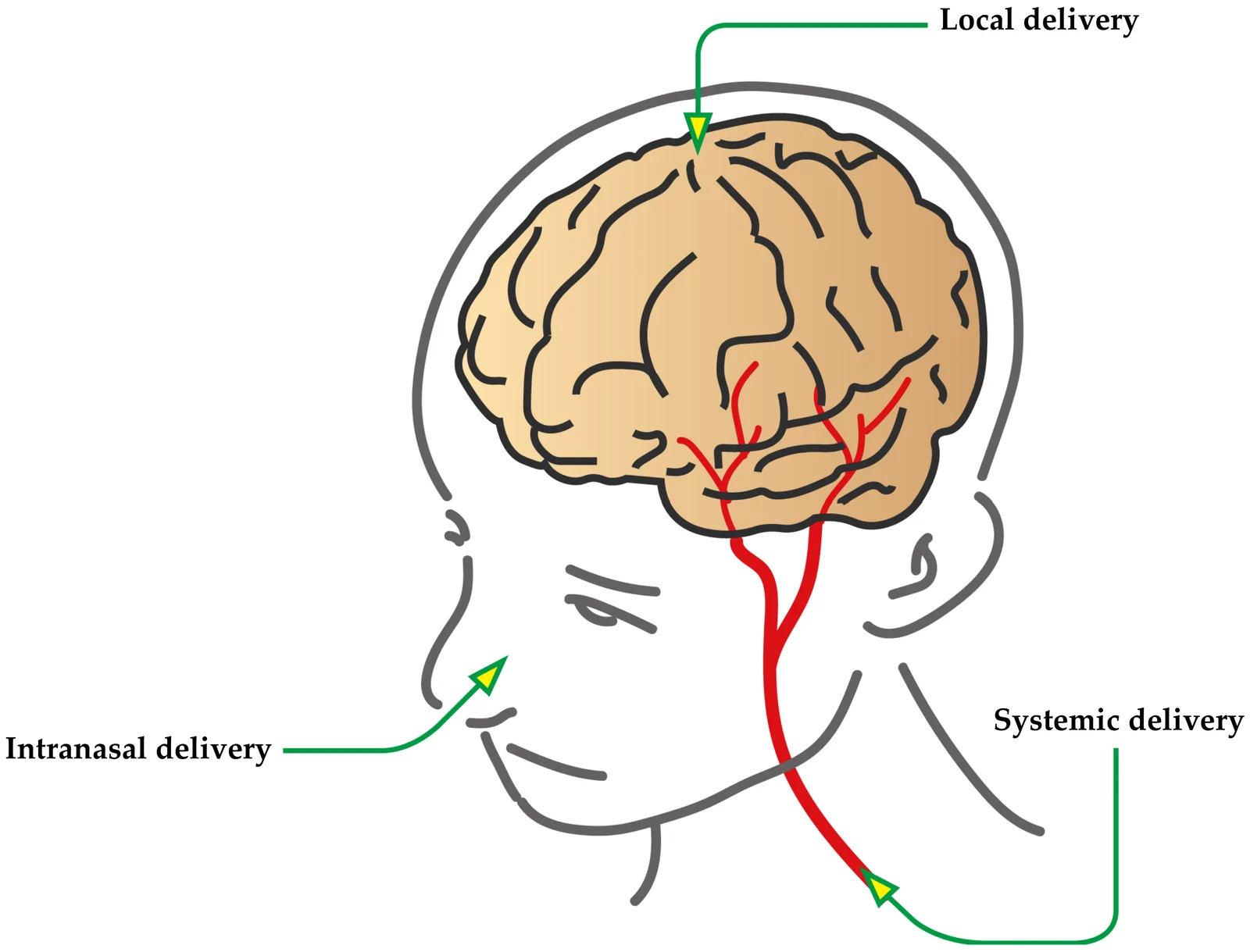
Different brain delivery strategies showing the major routes: local delivery, intranasal delivery, and systemic delivery.

Various strategies have been developed over time to deliver active therapeutic agents to the brain to treat neurological disorders (Figure 2). A best route, the drug formulation and optimal window of administration have been the fascinating subjects in the field of brain drug delivery for treatment of neurological disorders. Generally, drugs can be delivered to the brain by local brain site injection, or via a catheter or direct drug administration following invasive surgeries. Drug-loaded polymeric biodegradable materials can be implanted for the sustained release of the drug at a specific site of the brain in this type of delivery strategy [22,23]. The local delivery route is among the most invasive administration routes, although very effective in animals in delivering drug formulation to brain, will fail in clinical context when treating real human (variable physiology) patients due to rapid drug degradation and clearance. Hence, steady pharmacological effect is needed with less invasive ways of drug delivery. Intranasal route of drug delivery is also promising, allowing the drug to directly reach the brain by bypassing the BBB [24,25,26]. The active drug, loaded in some nanocarrier system after reaching the nasal cavity, directly reaching the brain through the olfactory pathway and trigeminal pathway [27,28,29]. Still, the intranasal route is not an ideal approach due to inconsistencies in the released dose at the target site, which is solely dependent on the nature of the nasal mucosa and its interaction with the drug [22,30]. To date, the systemic delivery route is the most studied and acceptable strategy to deliver the drug to the brain. However, the BBB is the main obstacle in this type of drug delivery strategy. A design must be created where the active drug can be loaded inside a nontoxic and permeable nanoparticles, which can cross the BBB [31]. Moreover, there are several approaches developed to increase the permeability of the BBB such as injection of hyperosmolar mannitol [32] causing reversible disruption, or by providing ultrasound as physical stimulus [33,34]. Either way, BBB disruption led to unavoidable influx neurotoxins causing significant damage to the brain [31]. Hence, advanced drug modification strategies could be helpful to increase the ability of drugs to cross the BBB in treatment of neurological disorders avoiding neuronal dysfunction due to BBB disruption. Lipid-based nanoparticles can pass the BBB in a safe and effective manner [35].

## 4. SLN as Advanced CNS Drug Delivery System

Although there are many drug delivery-approaches for the treatment of neuronal disorders, unfortunately, ultimate success has not yet been achieved, due to a lack of target specificity, lower bioavailability, and toxicity concerns. At present, the majority of them are trial-and-error-based, and far from complete. Recent studies are inclusive of the efficiency and relative safety of receptor-mediated drug delivery regarding nutrient uptake and the recognition of specific ligands modulating endocytosis. Drug molecules modified with carrier molecules such as nanoparticles and liposomes can be delivered by the unique process of “tricking”, where the receptors mediate internalization and ligands bind target cells in brain tissue [36].

In the context of carrier-mediated drug delivery, the reengineering of active drug and drug components together with carriers in pharmaceuticals that can penetrate the brain through the BBB are of interest to develop drugs that are effective in the treatment of neurological disorders. Some research findings showed that small lipophilic molecules of less than 400 Da can freely diffuse across the BBB endothelium [17]. Due to their size and properties, lipid nanoparticles, therefore, can interact as a drug carrier molecule with the BBB and its components, and cross the BBB. These factors will be considered in relation to drug discovery in general and CNS drug discovery in particular [37].

Over the years, nanoparticles have emerged as a suitable drug–carrier system for drug delivery in an effective and site-specific manner, due to their unique size and physicochemical properties, which enable them to cross various anatomical barriers. Furthermore, nanoparticle properties can be improved by enhancing their ability to penetrate through several anatomical barriers, releasing the drug content in sustained manner and maintaining their particle size. Most importantly, polymeric nanoparticles of a biocompatible and biodegradable nature are of essential interest for target-specific drug delivery [38]. To date, limited numbers of polymers have received regulatory approval for use in clinics. Cost-effectiveness is another barrier for the polymeric nanoparticles which limits their wide spread application [7]. Lipids have been put forward as an alternative carrier to surmount the limitations of polymerics during the formulation of lipophilic pharmaceuticals. Contrasting with the polymeric nanoparticles, lipid nanoparticles such as solid lipid nanoparticles (SLNs) are gaining considerable attention as a worldwide drug delivery system for various clinical purposes [39].

**Figure 3:**
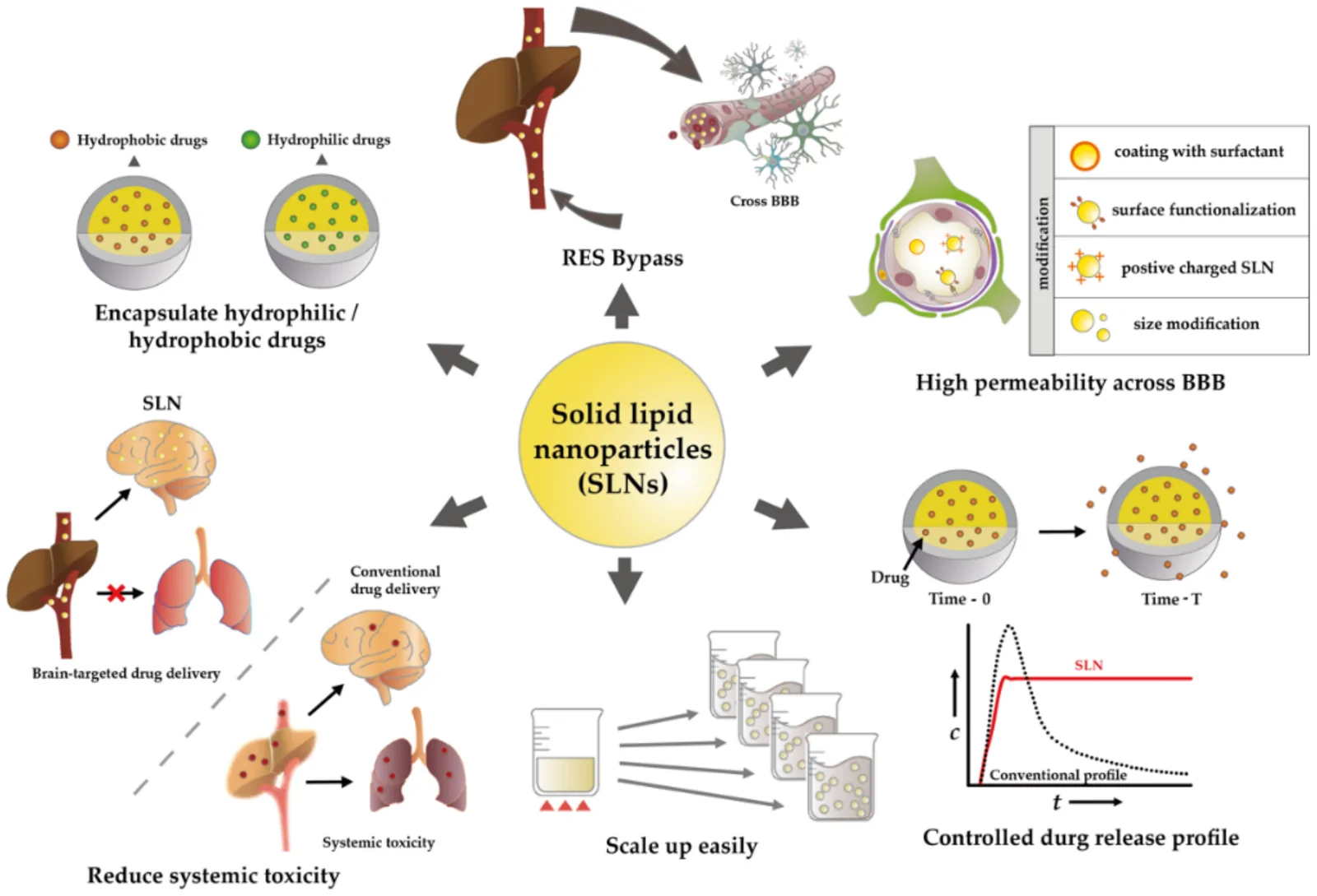
Highlighting the utilities of SLNs and their modifications such as: encapsulation of hydrophilic and lipophilic drugs, capable to cross the BBB for target specific drug delivery due to their unique physicochemical nature, can bypass RES system, reduce systemic toxicity, sustained or controlled drug release in a time dependent manner, and can be scaled up in a cost-effective way.

SLNs are unique lipid-based biocompatible nanocarrier systems mainly constituting lipid or modified lipid (triglycerides, fatty acids, or waxes) nanostructures (10–1000 nm diameter size range). SLNs have a solid hydrophobic lipid core, in which both hydrophilic and lipophilic drugs can be dispersed [40,41]. They play a significant role in crossing the reticuloendothelial system (RES) of the BBB [42,43,44]. This colloidal nano-carrier was developed as a better substitute for polymeric nanoparticles and liposomes due to its solid lipid composition instead of an aqueous solution, to safeguard active-drug-counteracting biochemical degradation [45]. As they are formed of a physiological solid lipid emulsion system by maximally avoiding organic solvents, they display better biocompatibility and reduced systemic toxicity in comparison to polymeric nanoparticles [46]. SLNs containing drug also show a sustained release feature due to the use of solid lipids, modified drugs, and additive ingredients in a particular ratio, providing a particular physicochemical state with the longest diffusion pathway and controlled drug release [47,48,49]. Some study findings show that the pharmacokinetics, tissue distribution, and bioavailability of SLNs loaded with nitrendipine (antipsychotic drug) could be improved by administering nitrendipine SLN in rats [50]. SLNs require a low cost for raw materials and production, have excellent physico-chemical stability, and can be commercially sterilized and lyophilized in an affordable manner. These features make SLNs advantageous for production on a large industrial scale [43]. Furthermore, SLNs serve as an ideal drug delivery system with various important characteristic features such as maximum drug bioavailability upon administration, specific tissue targeting, controlled release kinetics, minimal immune response, the ability to deliver traditional pharmaceutical formulations and biomolecules, sufficient drug loading capacity, good patient compliance, and cost effectiveness, which might make it a better and unique system in comparison to polymeric nanoparticles and other formulations. Some of the advantages and disadvantages associated with SLNs are mentioned in Table 1 and Table 2, with the utilities represented in the Figure 3.

### 4.1. Drug Stabilization by SLN

There are various drug formulations that, despite their ability to cross the BBB, show poor plasma stability, rapid clearance, and a short half-life, resulting in poor in vivo efficacy [70]. SLNs are one of the colloidal nanoformulations that can overcome the drug stability issues. SLNs’ drug delivery design could improve the drug-loading capacity, drug stability, and bioavailability after crossing the BBB, maintaining the drug plasma concentration with less possible toxicity. For example, the anticancer drug camptothecin can cross the BBB and be used in glioblastoma therapy [71,72], although it cannot be used in clinical protocols due to its poor stability. However, the formulation has been used to stabilize the physiological pH and further stabilize size, charge, and pharmacokinetics using SLN modifications, showing better brain targeting efficacy [49,73,74,75].

### 4.2. Enhanced Bioavailability of Drugs by SLN

When certain lipophilic oral formulations for the treatment of neurological disorders suffer from poor bioavailability due to the first pass metabolism, these can be improved by encapsulation in SLN. The permeability of the drug molecules to the brain site can be improved by BBB-active drug efflux transporters based on the efflux mechanism. Manjunath and Venkateswarlu [76] tried to improve the bioavailability of the antipsychotic drugs clozapine and nitrendipine through their various SLN formulations, using PEG and Tween 80. 5-fluoro-2′-deoxyuridine (FUdR) to the brain, 3′,5′-dioctanoyl-5-fluoro-2′-deoxyuridine (DO-FUdR) incorporated into solid lipid nanoparticles (DO-FUdR-SLN), has shown good brain-targeting efficacy for CNS disorders, shown in the studies by Wang et al. [77]. The mechanism behind the enhanced drug bioavailability could be explained by: (1) surface modification of the SLN with Pluronic F-68, resulting in a steric hindrance effect, which would further decrease the adsorption of opsonin onto SLN in the plasma; hence, RES uptake can be reduced prolonging the retention time in plasma; (2) higher concentration gradient at the brain capillary due to higher SLN load, resulting in enhanced transport across the brain endothelium followed by endocytosis and drug release from SLN. Another study showed that the plasma half-life of the drug noscapine was enhanced by surface PEGylation, which could escape RES uptake [78].

### 4.3. Enhanced BBB Permeability of Drugs by SLN

SLN and their modifications are considerably better carrier system, which can encapsulate a broad range of drug molecules to deliver the drugs in a safe and effective manner. The anticancer drug doxorubicin (a hydrophilic drug) cannot cross the BBB, and shows acute toxicity and cardiomyopathy, leading to severe issues, which sometimes limits its use in clinical context. These shortcomings can be avoided if it can reach the target site. A study showed doxorubicin being incorporated in SLN prepared from a warm oil-in-water microemulsion containing stearic acid as an internal phase, with Epikuron 200 as a surfactant and taurocholate sodium salt as a cosurfactant. SLN-modified doxorubicin upon i.v. administration to rats showed lower drug concentration in the liver, heart, and kidneys, while a higher concentration was shown in the brain due to RES escaping, in comparison to free doxorubicin [79]. In another study, the presence of a stealth agent with SLN loaded with doxorubicin was shown to increase the plasma circulation of drugs and drug concentration in the rat brain after i.v. administration [57]. The study on riluzole (a drug for amyotrophic lateral sclerosis) showed that the SLN formulation results in better drug delivery to the brain [80]. The SLN formulation of paclitaxel (anticancer drug) stabilized with Brij 78 has shown better drug distribution in the brain in comparison to naïve drug, probably followed by P-gp-efflux-mediated brain drug delivery [81].

**Figure 4:**
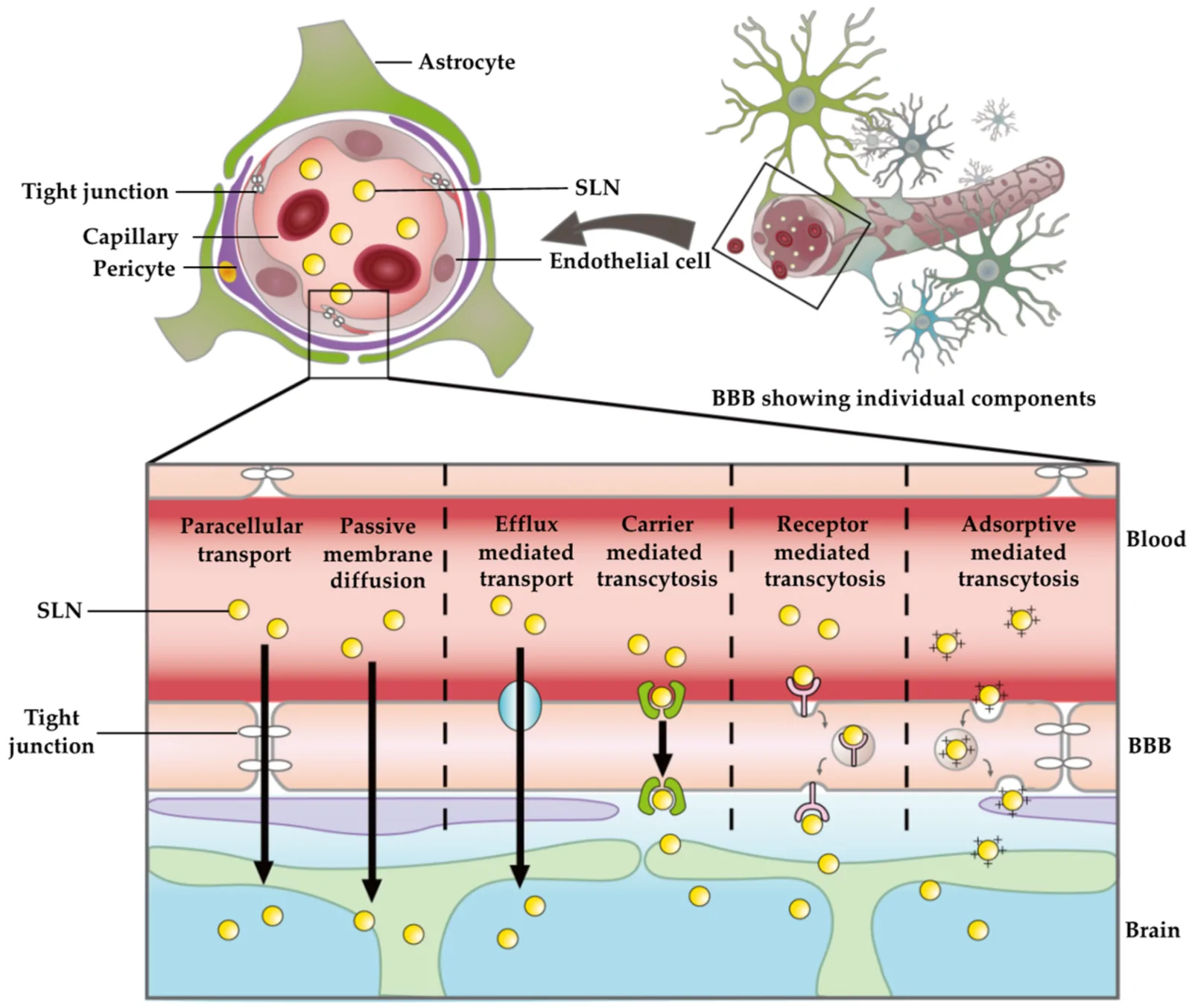
BBB showing individual components and how the drug loaded SLNs can cross the BBB by different physiological mechanisms such as: Paracellular pathway and passive transmembrane diffusion; Protein mediated transport; Receptor-mediated transcytosis (RMT); and Adsorptive-mediated transcytosis (AMT).

It is necessary to understand the different pathways through which the biological and pharmaceutical active components can cross the BBB (Figure 4). The detailed transportation mechanisms of each pathway are briefly described to obtain a general idea of how the active pharmaceutical components, with the help of a carrier system can cross the BBB to reach the brain. Although many strategies have been developed to nonspecifically disrupt the BBB, allowing the pharmaceutical agents to enter into the brain, these may also allow circulating toxins enter the brain from the blood, causing severe neuro toxicity. There is a need for an ideal and safe approach by which we can improve the brain permeability of the drugs in a target-specific and sustained-release manner, without disrupting the BBB. Recent SLNs, and the modifications in drug delivery for the treatment of neurological disorders, are very promising biomaterials in the context of BBB-targeted drug delivery.

(1) Paracellular pathway and passive transmembrane diffusion

The tight junctions between the endothelial cells play a major role in the paracellular pathways, allowing only hydrophilic molecules to pass. There is an alternative transcellular pathway, which allows lipophilic small molecules, with a molecular weight lower than 400 Da, to undergo transmembrane diffusion in a non-saturable and non-competitive manner [82]. Besides this, the endothelial cells of the BBB also restrict active molecules from entering the brain by limiting pinocytic activity [83,84,85].

(2) Protein-mediated transport

Transporter proteins are unique proteins responsible for delivering brain specific molecules by carrier-mediated transport and efflux proteins. Those are present on the luminal and basolateral side of the endothelial cells. For example, organic cation and anion transporters, GLUT-1, and large neutral amino acid transporters (LAT), play an important role in the delivery of various biomolecules [84,86,87] and their substrates in a size- and stereo-selective manner [88]. Pharmaceutical drug molecules and their modifications can also pass through the BBB using these efflux mediated proteins i.e., P-glycoproteins (P-gp or ABCB1, MDR1 gene product), breast cancer resistance proteins (BCRP/ABCG2), and the multidrug resistance-associated proteins (MRP1, 2, 4, and 5, ABCC) [84].

(3) Receptor-mediated transcytosis (RMT)

RMT-mediated transport is a specialized transport system by which endogenous molecules can cross the BBB for brain delivery by the activation of peripheral brain endothelial cells [89]. This process is one of the one of the most promising pathways for drug delivery through the BBB. The steps involved in this process are as follows: endocytosis, intracellular vesicular trafficking and exocytosis [90]. During this RMT process, active components bind to their specific receptors on the luminal side of the endothelial cells. Then the receptor–ligand complexes build up intracellular transport vesicles. The formed vesicles cross the cell to release the ligand to its basolateral side using exocytosis [91]. The major receptors involved in RMT are transferrin receptor (TfR), insulin and insulin like growth factor receptor, low-density lipoprotein receptor (LDLR), low-density lipoprotein -receptor-related protein 1 and 2 (LRP1 and LRP2), scavenger receptor class B type I (SR-B1), leptin receptor, albumin receptor [90,92], and lactoferrin receptor [88,91]. The endocytic vesicles, which play a major role in this RMT, are clathrin-coated pits, caveolae, and macropinocytosis vesicles. Clathrin-coated pits are involved in most of the internalization processes that are mediated by receptors such as TfR or insulin receptors [89,90]. Hence, RMT is dependent on the internalization pathway (clathrin-mediated or caveolae) and the type of ligand binding to the receptors [89].

(4) Adsorptive-mediated transcytosis (AMT)

AMT is another important BBB-crossing pathway, without the involvement of specific plasma-membrane receptors. Hence the binding affinity of AMT is low, but the binding ability is high, with the same level of transcytosis efficiency as RMT [93,94]. The basic mechanism of AMT is based on the electrostatic interaction between the charged molecule, such as the positively charged protein and negatively charged luminal membrane of the brain endothelial cells [95,96].

## 5. Methods to Improve SLNs for Brain Drug Delivery

**Figure 5:**
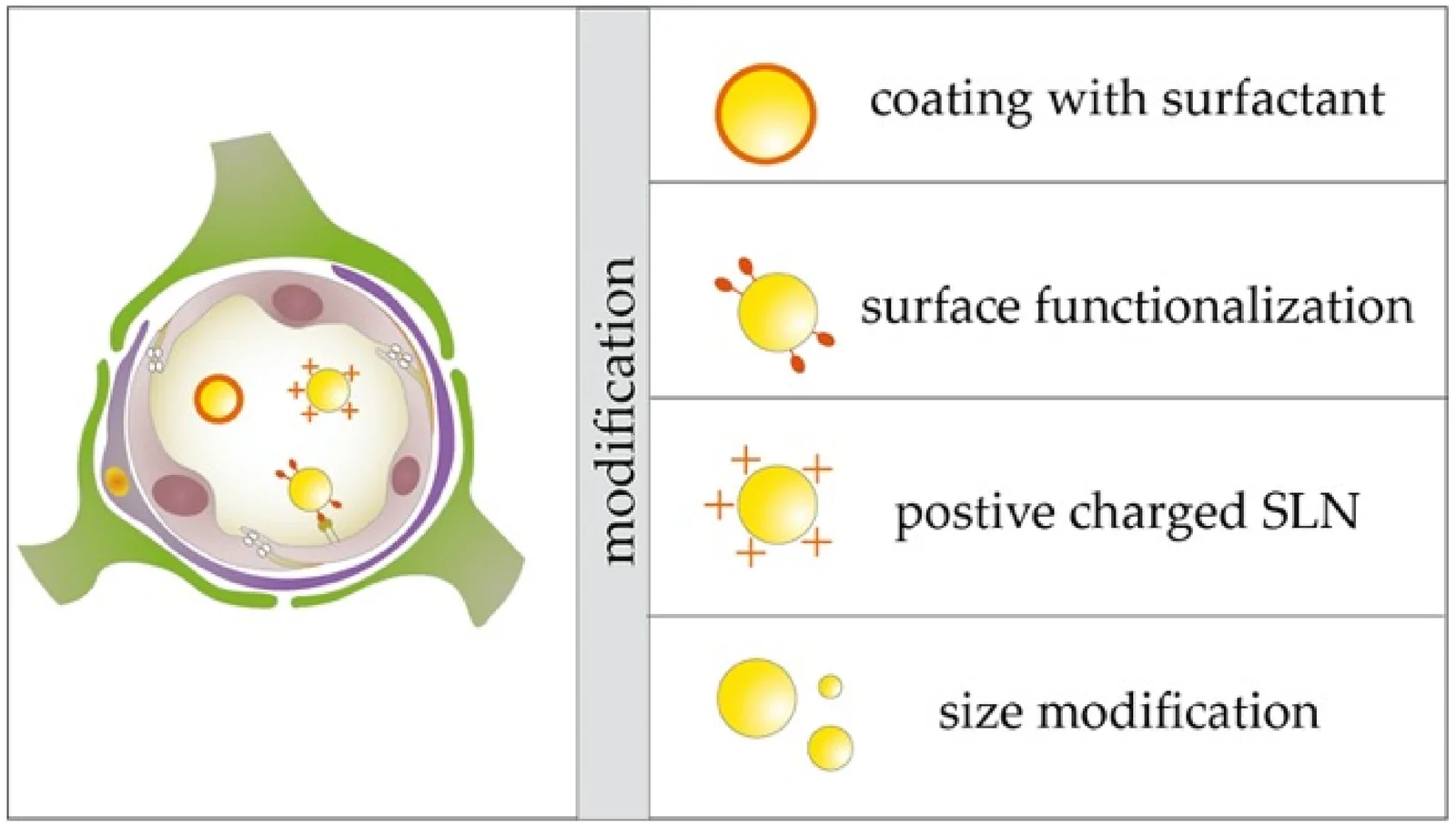
Methods to improve SLNs for brain drug delivery: coating with surfactant; surface functionalization; cationization; size modification.

There are various characteristic features which can be modified to improve the quality of the SLNs. For example, the SLNs loaded with drugs such as paclitaxel, vinblastine, camptothecin, etoposide, and cyclosporine (hydrophilic drugs) [41,49,73,97], due to their unique size, surface hydrophobicity, and surface mobility, can cross the BBB. However, those drugs may have shown limitations in their pharmacokinetics, resulting in poor pharmacological activity and therapeutic efficacy. The reason for this is their detection by the reticuloendothelial system (RES). Recent research has focused on how to improve the shortcomings associated with SLNs. Some of the approaches used are briefly described here (Figure 5).

(a) Particle size

Primarily particle size plays a major role in SLN efficacy during drug delivery, including in the therapeutic effect achieved and clearance from the body. The SLNs should be small enough or, if it is able to deform itself, it can avoid the splenic filtration process at the interendothelial cell slits (IES) [98,99]. The endothelial cells’ slit width from 200 to 500 nm in width [98]. Hence, for the long-term circulation of the SLNs, they should be modified in such a way that the particle size does not exceed 200 nm, so that they have an increased blood circulation time and the drug in contact with the BBB for the maximum time. This would lead to better brain drug delivery across the membrane. Sometimes if the size of the SLNs is larger, it can be deformed adequately to bypass IES filtration. 

(b) Surface coating with hydrophilic polymers/surfactants

As RES-mediated active detection of colloidal nano-particles is very common, they can be cleared very quickly by the liver after the administration significantly reduces the half-life of the drug. Opsonization plays a major role in the entire process of drug clearance. RES recognition of the drug component should be prevented to increase its efficacy [100]. SLNs’ modification, by coating them with a suitable surfactant or hydrophilic polymer, can bypass RES recognition and escape phagocytosis. If the hydrophobic nature of the nanoparticle containing the drug can be shielded, it will be sterically stabilized, thus avoiding opsonization, and further increasing the blood circulation time and hence the bioavailability [101]. For example, SLNs coated with a hydrophilic polymer polyethylene glycol (PEG) showed encouraging results due to their hydrophilicity, chain flexibility, electrical neutrality, and lack of functional groups, preventing them from undesired interactions with the biological components. Furthermore, when PEGs with a molecular weight of 2000~5000 are coated on SLNs, plasma protein adsorption can be reduced, and the thicker the PEG coat, the slower the RES clearance. This leads to better protection against first pass metabolism [41,102,103]. Other examples of hydrophilic compounds such as Brij 78, Poloxamer F68, and Brij 68, when coated with the paclitaxel-SLN formulation, show significantly increased drug bioavailability during i.v. injection in comparison to the plain drug [97]. Polysorbate (20, 60, 80)-coated SLNs showed an enhanced pharmacological effect through its easy transportation across the BBB via endocytosis triggered by apolipoprotein [103,104]. Another study reported that plain and stealth SLNs of doxorubicin and pristine can cross the BBB, without any functionalization. However, stealth SLNs showed better brain delivery of doxorubicin than pristine SLNs. Better results were found with increasing amounts of stealth agents, resulting in a longer blood circulation time in case of SLN-doxorubicin stealth [57,105,106,107]. 

(c) Use of ligands

Ligands increase the selectivity of SLN to make it a long-circulating carrier system and act as a homing device, which may specifically bind to surface receptors expressed by certain cell types, such as folic acid (over-expressed in cells of cancers with epithelial origin), low-density lipid (LDL) (B16 melanoma cell line shows higher expression of LDL receptors), and peptide receptors (such as somatostatin analogs, vasoactive intestinal peptide, gastrin-related peptides, cholecystokinin, gluteinizing hormone releasing hormone), leading to increased selectivity [108,109]. Ligand-conjugated SLNs show a higher retention of drug molecules at the BBB [60]. Another study showed that colloidal particles coupled to sterically stabilized cationized albumin have a better interaction with brain endothelial cells and higher intracellular accumulation [110].

(d) Conjugation of SLN with arginine-glycine-aspartic (RGD)

Studies have showed that doxorubicin-SLN, when conjugated with RGD, increased in vitro antitumor efficacy and in vivo cytotoxicity in a target specific manner in comparison to non-targeted SLN [111]. RGD-conjugated SLNs can easily cross the BBB easily CNS drug delivery to the brain. One study showed that a SLN formulation of docetaxel functionalized with angiopep-2 can specifically bind to the LDL-receptor-related protein 1 (LRP1), which is overexpressed at the BBB, showing higher in vitro cytotoxicity and BBB permeability by receptor mediated endocytic processes [112]. Other studies are based on how to attaching a targeting ligand to an SLN, such as by linking a fatty acid of the NP to an amino group of the ligand [113], an amino group of a phospholipid to an acid group of the ligand [114], or an amino group of the chitosan coating to an acid group of the ligand [115]. These could lead to better brain drug delivery by the SLNs across BBB.

## 6. SLNs Different Models

**Figure 6:**
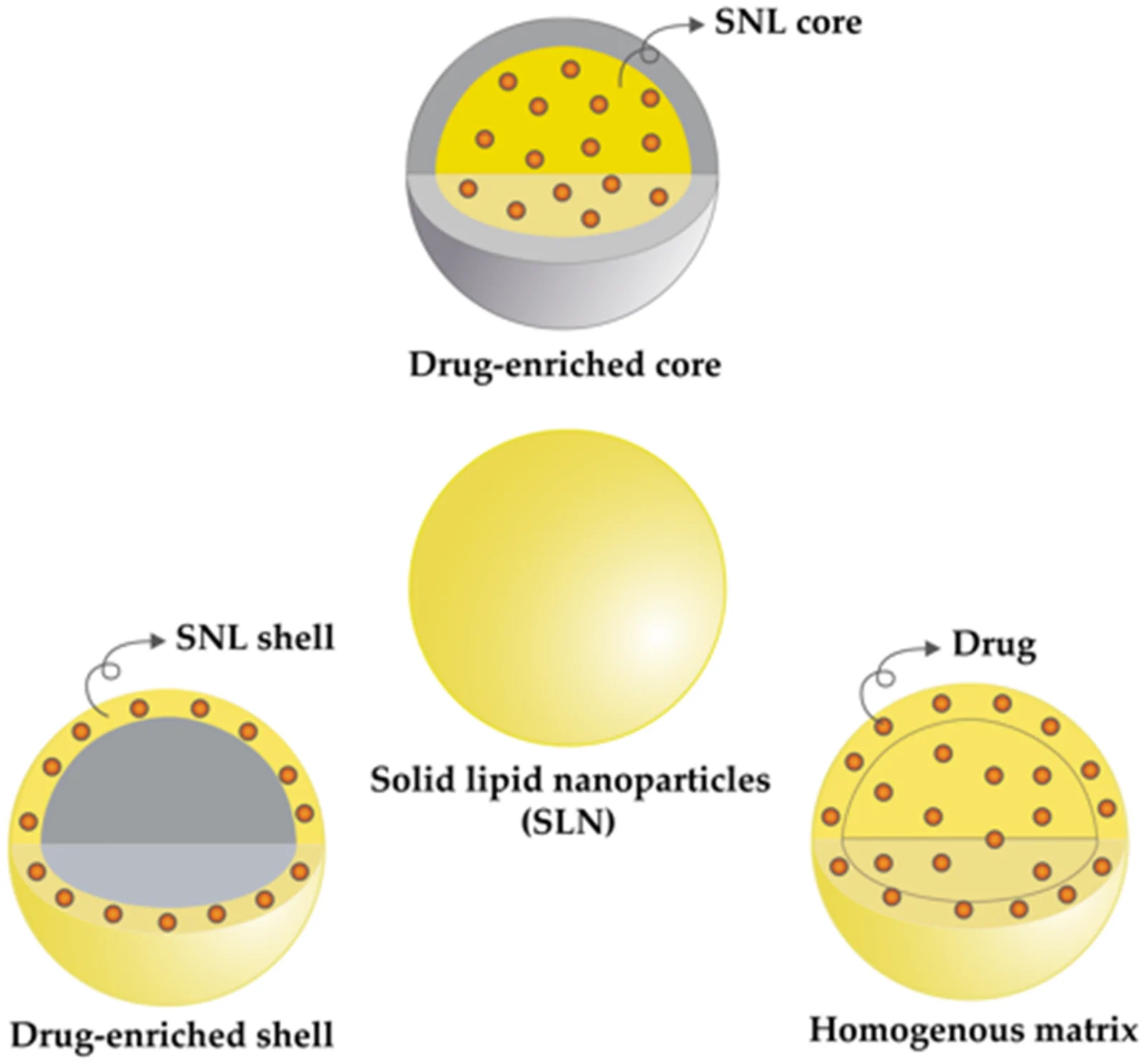
Different representative models for SLNs showing the drug distribution in the lipid core: Drug enriched core model, Drug enriched shell model, Homogenous matrix model.

There are various patterns of SLN formulations according to the distribution of drug components within them. Different models of SLNs are as follows (Figure 6)

### 6.1. Drug-Enriched Shell Model

In this model, the core of the SLN is drug free. The main active drug is distributed around the shell, as shown in Figure 6. A hot homogenization process is used for synthesis of this type of SLN. Hence, only the lipid content precipitates at the core at a recrystallisation temperature, leaving behind the drugs at the outer shell upon a decrease in the temperature of the obtained dispersion. The resulting solid lipid core is formed by recrystallization of the lipid independent of the drug component. In this types of SLN, the burst release of drug particles happens faster due to the large surface area of the outer layer where the drug is deposited [116,117]. However, the burst release can be controlled by replacing the smaller drugs with larger ones, such as lipid microparticles, and depending upon the properties of the surfactants used during formulation [118]. The use of surfactants at a lower concentration during the synthesis of SLNs could control the burst and, hence, prolong drug release.

### 6.2. Drug-Enriched Core Model

In this model of SLNs, the active drug is concentrated at the core with the outer lipid shell. This type of SLN is formed in several steps. The first liquification of drug in the lipid is carried out, leading to saturation solubility for the formation of drug lipid emulsion. Then, the mixture is cooled, and the active drug is concentrated at the center due to supersaturation. After further cooling of the dispersion, the lipid content is recrystallized as an outer layer containing the active drug at the core as shown in Figure 6. During this type of SLN formation, the drug precipitates prior to crystallization of the lipid; hence, it concentrates at the central core, surrounded by the lipid outer layer. In this type of SLN model, drug release is controlled by the nature of the lipid membrane, followed by Fick’s law of diffusion [43].

### 6.3. Homogeneous Matrix Model

In this model, the active drug content is distributed in the lipid matrix of the SLNs Figure 6. This model is also known as the solid solution model. The drug may be present in dispersion or in amorphous clusters in the lipid. This type of SLN is prepared by a cold homogenization process, where the drug and the lipid interact. The drug has strong molecular interactions with the lipid to form this type of SLN. Generally, lipophilic drugs are encapsulated in the lipid matrix during the preparation of this type of model SLN, without the use of surfactants. The drug release profile is extended in this type of SLN formulations, due to the firm molecular dispersion of drug particles in the colloidal matrix [116].

## 7. Synthesis Procedures for SLN

The main component precursors required for SLN synthesis are emulsions, microemulsions, and micellar solutions [119], which include solid lipid (beeswax, stearic acid, cholesterol, caprylic/capric triglyceride, cetylpalmitate, glyceryl stearate (-mono, and -tri), glyceryl trilaurate, glyceryl trimyristate, glyceryl, behenate (compritol), glyceryl tripalmitate, hardened fat (witepsol E85, H5 and W35), monostearate monocitrate, solid paraffin, behenic acid triglycerides, partial glycerides, fatty acids, steroids, and waxes), surfactant (stabilizes the lipid dispersion, Ex. phosphatidyl choline, soy and egg lecithin, poloxamer, poloxamine, polysorbate 80), and water [120], along with cosurfactants (sodium dodecyl sulphate, tyloxopol, sodium oleate, taurocholate sodium salt, sodium glycocholate, butanol)preservatives (thiomersal), cryoprotectants (gelatin, glucose, mannose, maltose, lactose, sorbitol, mannitol, glycine, polyvinyl alcohol, polyvinyl pyrrolidone), and charge modifiers (dipalmitoyl phosphatidyl choline, stearylamine, dicetylphosphate, dimyristoyl phophatidyl glycerol) [121].

When administered, the biological efficacy of the drug-entrapped SLN depends upon its physicochemical properties, such as its size, shape and chemical nature. These formulations are amenable to change, depending on the requirement. Aiming to obtain variation in the nature, size, and shape of SLNs, many techniques have been developed over the years. Some of the major techniques used are mentioned here and all the procedures are shown in Table 3.

### 7.1. High-Shear/High-Speed/-Ultrasonication Homogenization

**Figure 7:**
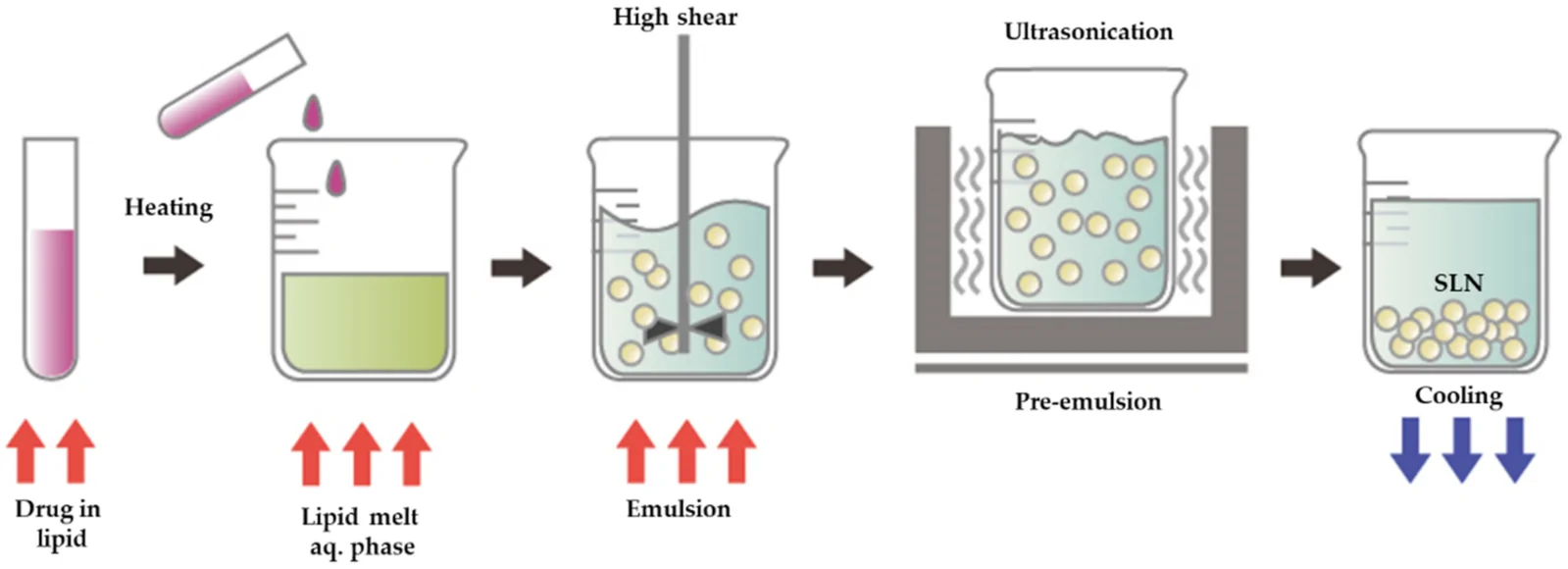
The procedure of high shear/high speed/ultrasonication homogenization.

High-shear homogenization was traditionally used for SLN [170] suspension. Melt emulsification produces SLNs using the high-speed/high shear homogenization method [169,171]. The nature of the SLNs (i.e., size, zeta potential, poly-dispersity index) produced depends upon various process parameters, such as time, stirring speed, and cooling condition. (Olbrich et al.). Ex-Witepsol W35 SLN dispersions [172] are produced by this method. Microparticle formation may be a limitation during this procedure affecting the dispersion quality. SLNs can also be synthesized by ultrasonication [120,170,173] (Figure 7) in an easily assessable way, to overcome the limitations of high-shear homogenization. The advantage of this process is that it does not need any sophisticated equipment. Its limitation may be the physical instability of the particles up on storage, and metal contamination. Hence, a combined process with a high temperature and high speed, along with ultrasonication, may ensure the production of quality SLNs [174].

### 7.2. Hot Homogenization

**Figure 8:**
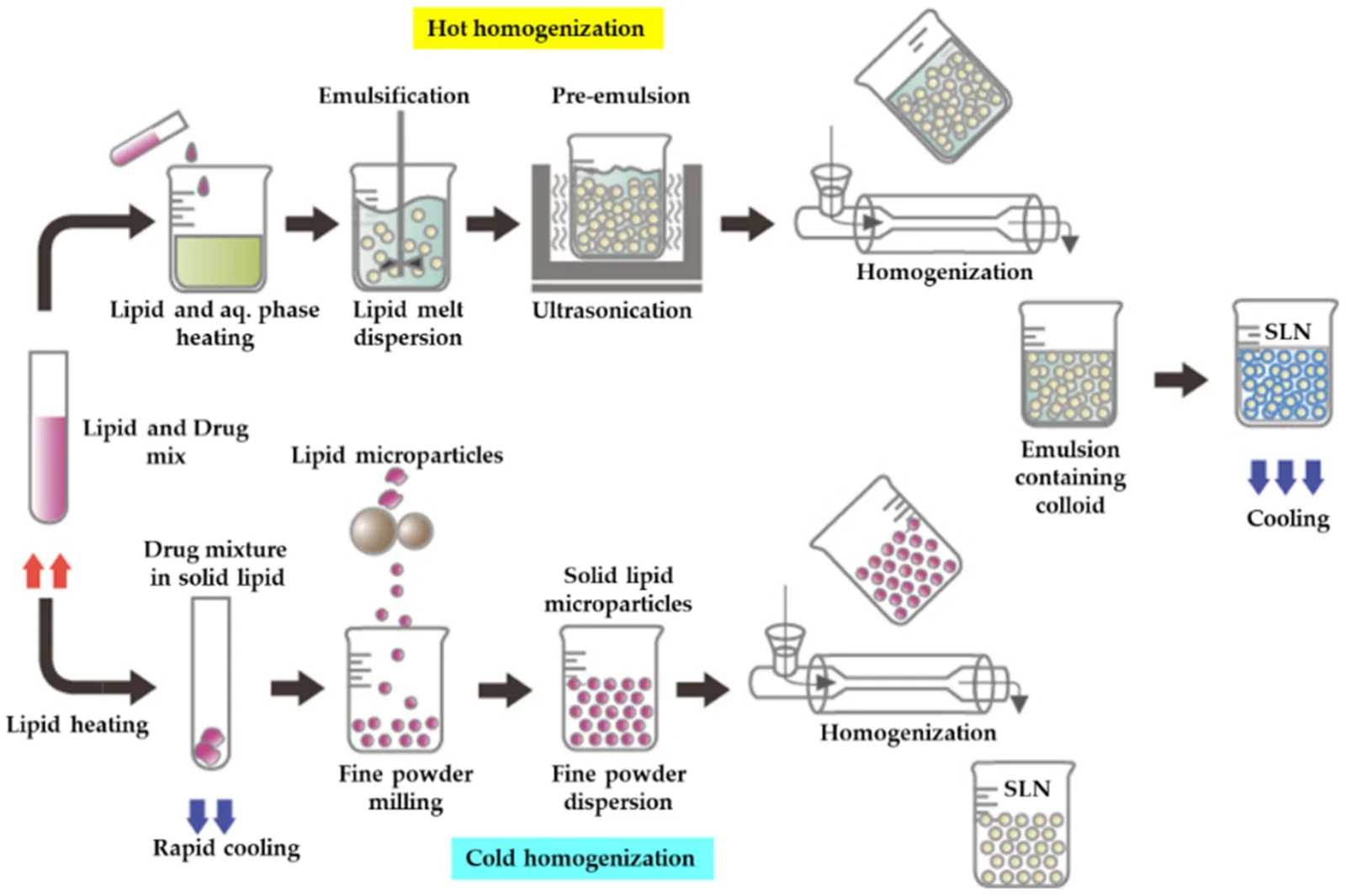
The procedure of hot homogenization and cold homogenization.

Hot homogenization is the process (Figure 8) of emulsion formation from lipids where, a high temperature (above melting point) is required to homogenize the lipid. During this process, a pre-emulsion (containing the drug and lipid mixture) is melted to obtain the aqueous emulsion by high-shear mixer. Then, this is cooled to obtain crystalized lipid SLNs as the final product. The final SLNs’ emulsion size and properties are dependent on the pre-emulsion contents and surfactant used. Microparticles are usually obtained during this process. However, at higher processing temperatures lower particle sizes are obtained, due to the reduced viscosity of the lipid phase at high temperatures [175]. High temperatures may be responsible for degradation of the drug and carrier substance, forming a limitation of this process [176].

### 7.3. Cold Homogenization

Cold homogenization (Figure 8) includes the cooling (dry ice or liquid nitrogen) of drugs and lipids to form a suspension at elevated pressure at a regulated temperature [176]. Then the solid drug–lipid core is ground by ball/mortar milling to obtain lipid microparticles of the size range between 50–100 mm. Sudden cooling may be a limitation of this process making the lipid fragile. However, it is a better option than hot homogenization, producing a broad range of SLN sizes [177].

### 7.4. SLN Prepared by Solvent Emulsification/Evaporation

**Figure 9:**
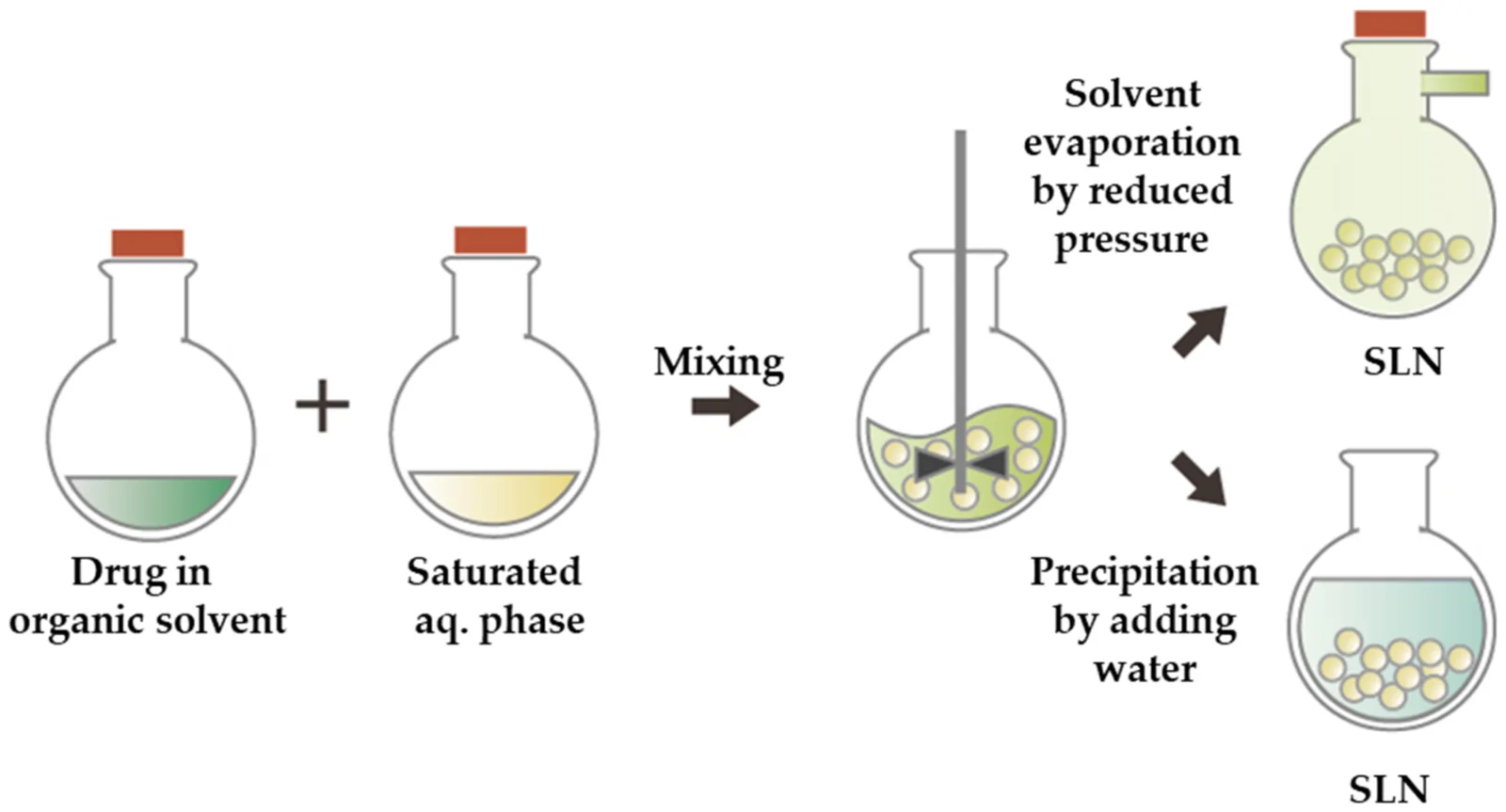
The procedure of emulsification solvent diffusion/evaporation.

During this process (Figure 9), a uniform lipid solution is prepared with a suitable organic solvent. Then water is added to this lipid solution to form an o/w coarse emulsion using a high-speed homogenizer. Then, a high-pressure homogenizer is used to obtain nano-emulsion of the solution mixture containing globules of a larger size. Then, from the nano-emulsion, the SLNs can be separated by continuous stirring overnight and separation of the organic solvent. After this, the lipid precipitate recovering in an aqueous solution is filtered to obtain the final SLNs, with a particle size of around 25 nm [178].

### 7.5. Micro-Emulsion-Based SLN Preparations

**Figure 10:**
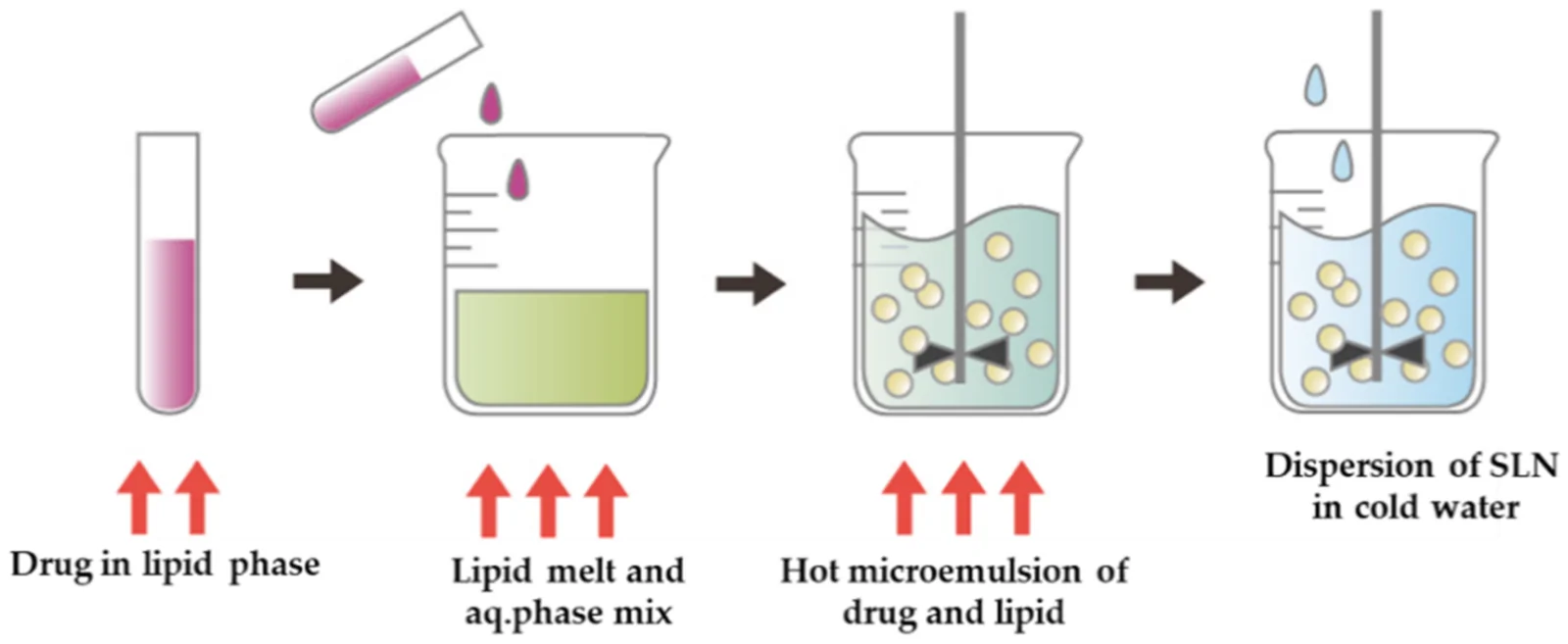
The procedure of microemulsion technique.

During this process (Figure 10), indirect heating is used to prepare the solid lipid melts. The aqueous solution of the solid lipid melts is then prepared using water, surfactant, and a co-surfactant. If these aqueous solutions are mixed with the lipid melt by continuous stirring, microemulsions are formed spontaneously. This process was first used by Gasco and co-workers for SLN formation based on the dilution of microemulsions [144]. The particle size depends on the solvents, e.g., larger-sized SLNs are obtained with more lipophilic solvents, whereas the use of hydrophilic co-solvents results in a small and uniform particle formation [179].

### 7.6. SLN Preparation by Using Supercritical Fluid

**Figure 11:**
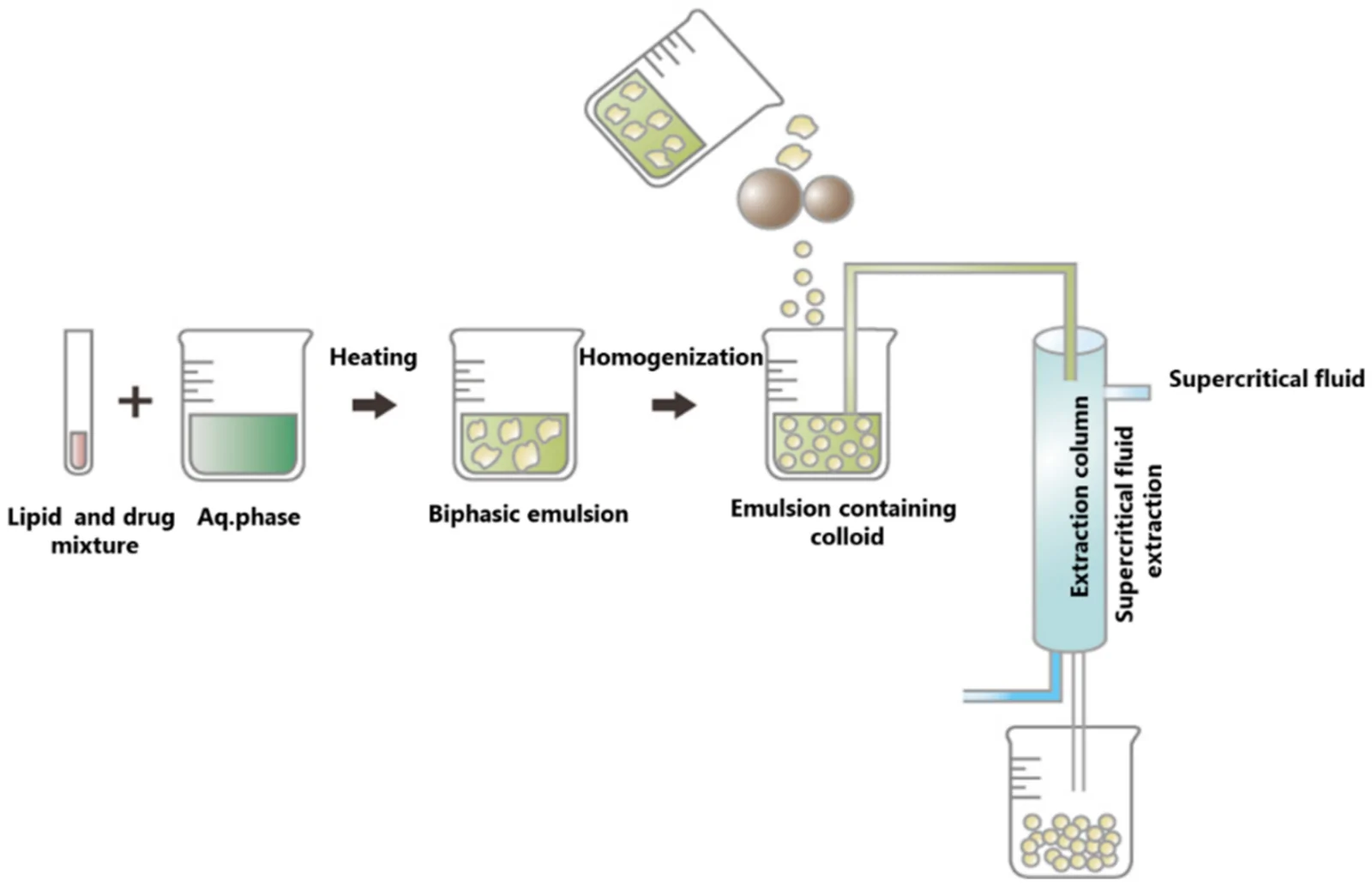
The procedure of supercritical fluid technique.

This process (Figure 11) is an advanced technique compared to the conventional methods where SLNs can be produced by particles from gas saturated solutions (GSS), with a tuning pressure and temperature based on solvent power, liquid-like densities, and gas-like transport properties. Lipid material is first melted by GSS. Then under ambient pressure, the lipid melt along with GSS will be dissolved in the super-critical fluid (SCF). This saturated solution mixture is then sprayed through an atomizer, where the SCF escapes rapidly, leaving fine dry SLNs. The main advantage of this technique is that it is a solvent-less process [180,181], where the powder form of the SLN in a nano-size range is formed. Ex-SLN can be prepared in the presence of carbon dioxide (99.99%), then used as a solvent during the rapid expansion of the supercritical carbon dioxide solutions (RESS) method [182].

### 7.7. Spray Drying Method

**Figure 12:**
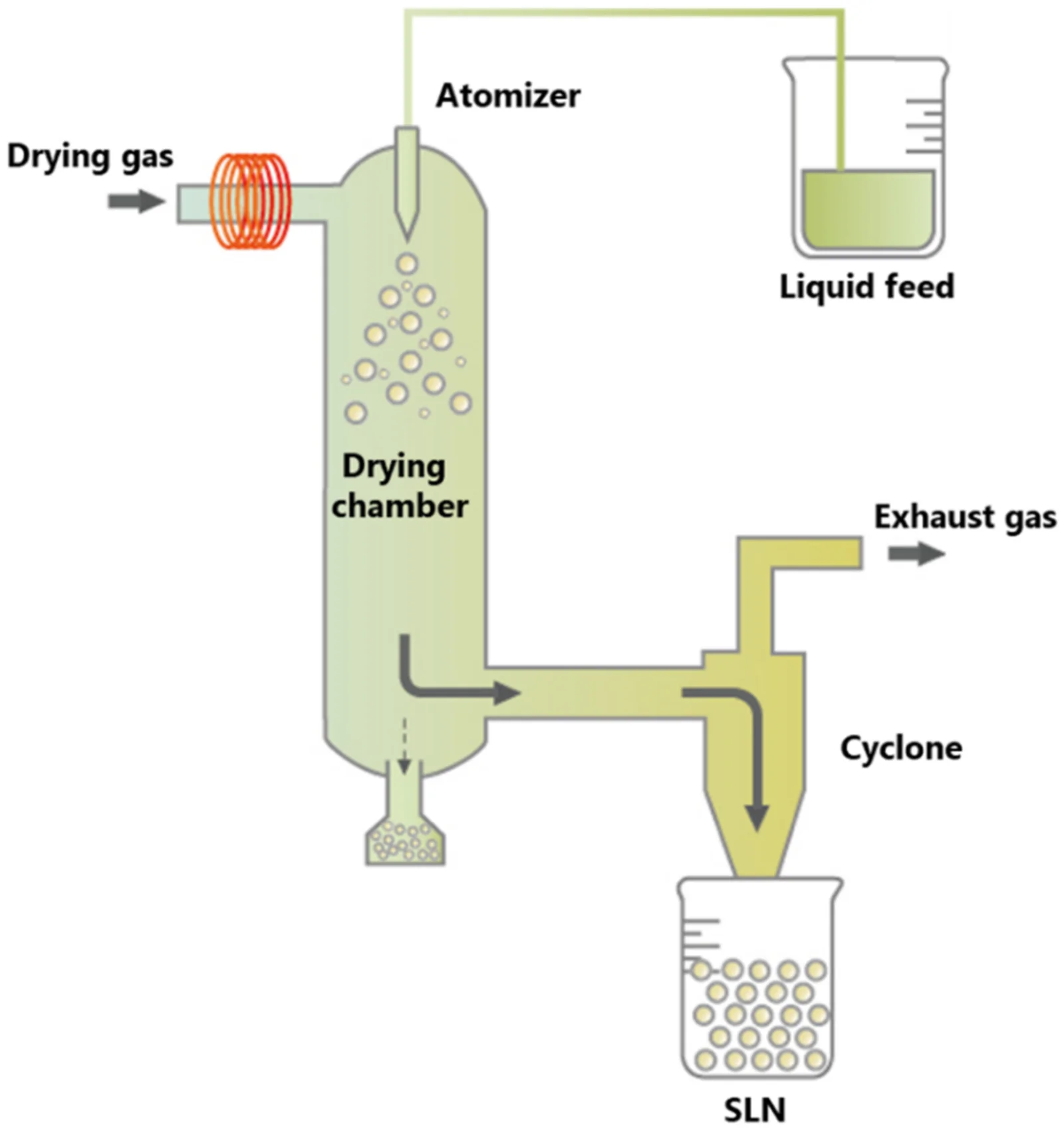
The procedure of spray drying technique.

This method is rarely used at present. This process is an optional and cheaper lyophilization method if there is a need to transform an aqueous SLN suspension into a drug product (Figure 12). Particle aggregation is the main limitation of this process, which occurs due to the high temperature, shear forces and partial melting of the particle [129]. This method is further restricted to lipids with a melting point above 70 °C [183,184].

### 7.8. Double Emulsion Method

**Figure 13:**
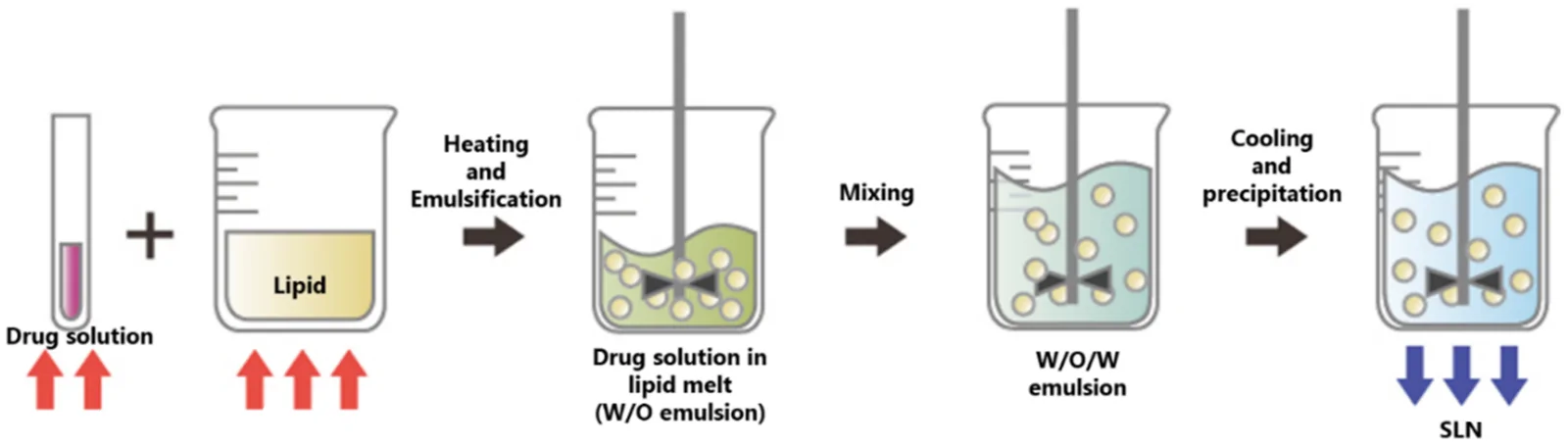
The procedure of double emulsion technique.

Generally, hydrophilic drugs can be loaded to SLNs by this double emulsion method (Figure 13), where solvent emulsification-evaporation is the core mechanism [185]. During this process, the drug is encapsulated with a stabilizing agent to prevent drug partitioning to the aqueous phase of w/o/w double emulsion. The double emulsion technique is the most frequently used technique. However, it produces larger-sized SLNs, which may need surface modification during synthesis [186]. 

## 8. Applications of SLNs in CNS Disorders

As the maximum of the pharmaceutical formulations for the treatment of CNS-related disorders cannot cross the BBB, there are limited therapeutic benefits. Accordingly, SLNs are one of the best rational biomedical approaches as they can significantly overcome BBB-associated limitations, leading to successful drug delivery. Recent research on SLNs as a carrier system have aimed to target the brain, delivering drugs across the BBB. SLNs, with its advanced features, is a new smart drug delivery system for the treatment of neurological disorders, with ideal characteristic features such a nanodiameter range, site-specific targeted delivery (via receptor-mediated transcytosis across brain capillary endothelial cells), physical stability, ability to escape the reticulo-endothelial system, extended blood circulation time, sustained release, and nontoxic, biodegradable, and biocompatible qualities. From an economic perspective, SLN manufacture is scalable and cost-effective [187].

**Figure 14:**
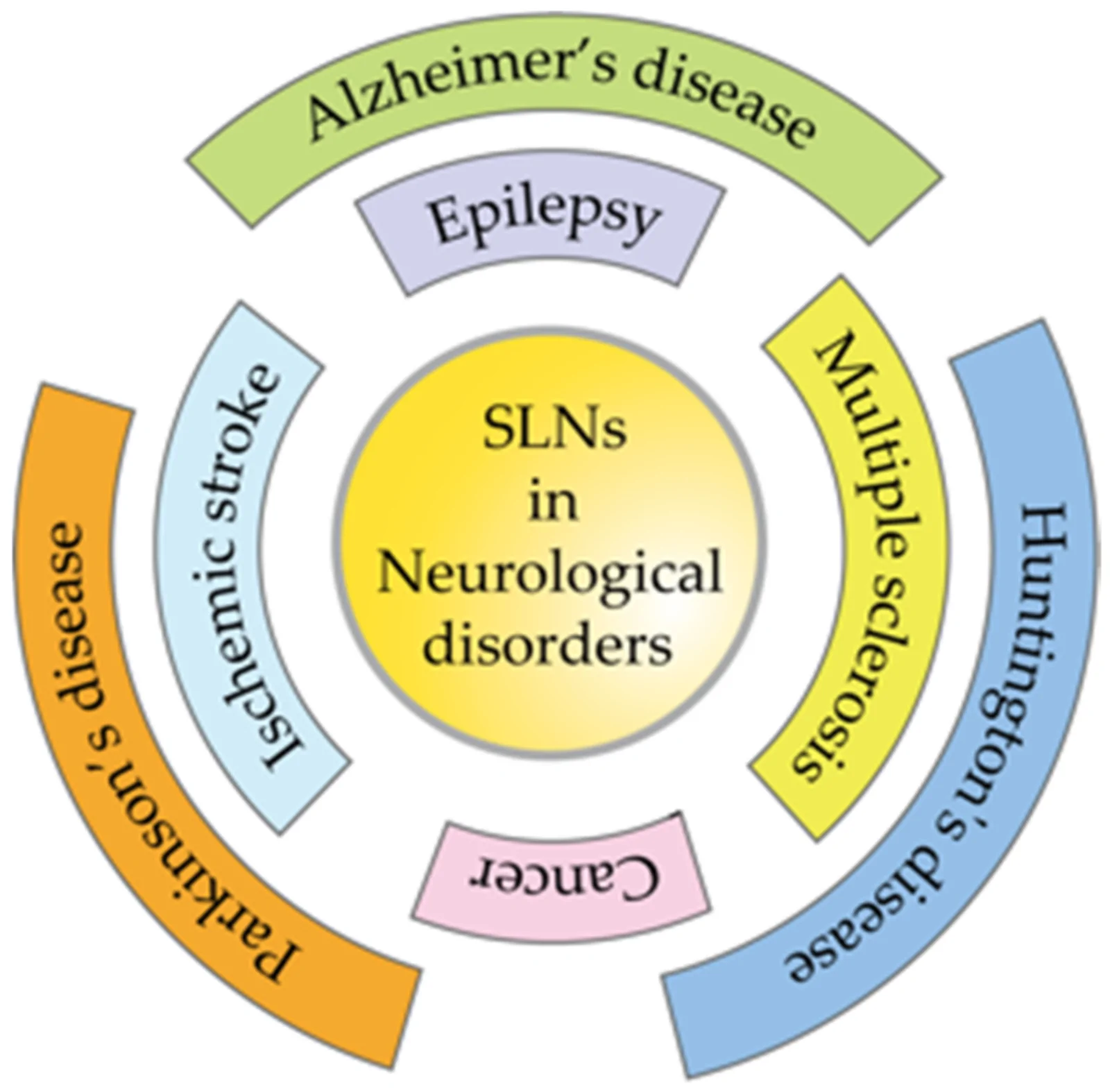
Representation of SLNs applications in CNS in various types of Neurodegenerative disorders.

Research has shown that there is a comparatively high accumulation and targeting potential for drug-loaded SLN carriers in brain than in other organs during intravenous administration [49]. SLNs, as unique delivery systems encapsulating active pharmaceuticals for the treatment of CNS disorders, can be delivered via oral, inhalational, and parenteral routes [188] to reach the neuronal sites. Thereafter, SLNs intervene in pathological signaling pathways as well as in the metabolism, correcting the neuropathologies. There are several prospective applications of SLNs loaded with drugs to treat CNS disorders. In recent years, many research studies have been published, and are ongoing, which are relevant to the use of drug-loaded SLNs for the treatment of various CNS disorders, including AD, PD, HD, multiple sclerosis, brain tumors and cancer, epilepsy, ischemic stroke, and certain neurodegenerative disorders (Figure 14). Some of these are provided in Table 4.

### 8.1. Drug Loaded SLNs for Alzheimer’s Disease

AD is a progressively degenerative neuro disorder mainly affecting the aged society. It is characterized by frequent cognitive function loss such as loss of memory and frequent behavioral change, leading to death [218]. Its therapeutics are based on targeting cholinergic dysfunction in developing cholinesterase inhibitors [219]. Donepezil, galantamine, and rivastigmine are the FDA-approved drugs use as acetylcholinesterase inhibitors to treat various grades of AD [220]. However, the required drug concentration cannot be reached at the site of the brain, which is one of the major limitations of these drugs. The main cause of this is the inability of the drugs to cross the BBB, minimizing the pharmacological effect. Higher drug concentrations need to be reached to provide better neuroprotection. SLNs, as an advanced drug delivery approach, have been used to load the existing drugs, further improving bioavailability and therapeutic efficacy in treating AD [221,222]. Donepezil (an anti-Alzheimer’s drug), when tailored to ApoE-targeted and SLN-based formulations, the in vitro study findings showed that it has enhanced drug delivery with a favorable release profile in CMEC/D3 brain endothelial cells and human SH-SY5Y neuronal cells [223]. SLNs loaded with galantamine hydrobromide is one of the most potent anti-AD drugs [192]. This drug composite has been synthesized by the solvent emulsification–diffusion technique, using Tween 80 as a surfactant. The resulting SLN has a particle size (772 ± 20 nm), polydispersity index (PDI; 0.432), and Z-potential (14.8 ± 3 mV). Piperine loaded in SLN is another anti-AD drug studied in vivo by Yusuf et al. [194]. This SLN is synthesized by the solvent emulsification–diffusion technique using glycerol monostearate as a common solid lipid and Polysorbate-80 coating for specific brain targeting. Kakkar, et al. studied the curcumin loaded in SLN, synthesized by Compritol888, Polysorbate-80, and soy lecithin. This was employed in the microemulsification technique for the treatment of [198] aluminum-induced AD. The specialized SLN can overcome the poor absorption, instability at physiological pH, rapid metabolism, and systemic elimination of drugs [216,224,225,226], improving the AD treatment strategy. The functionality of this, and related studies on SLNs loaded with nicotinamide, sesamol, galantamine, quercetin, piperine, ferulic acid, epigallocatechin3-gallate and curcumin, is briefly illustrated in Table 4.

### 8.2. Drug Loaded SLNs for Parkinson’s Disease

Parkinson’s disease (PD) is the second most common neurodegenerative disorder, after AD. It involves symptoms of psychological disorders, depression, tremor, and bradykinesia [227] with advancing age [228]. The pathological mechanism includes the progressive loss of the dopaminergic neuron as a result of mitochondrial dysfunction, oxidative stress, and protein misfolding. Levodopa is the best drug of choice for PD to date [229,230,231], targeting the dopaminergic receptor. Levodopa is able to cross the BBB. However, the required drug bioavailability is unsatisfactory [232,233], with lower therapeutic efficacy. The recent SLN drug delivery approach synthesized by the microemulsion technique was introduced to encapsulate the levodopa to overcome the limitations [199]. Bromocriptine loaded in SLNs synthesized by ultrasonication and homogenization (with a mean diameter of 197.5 nm, PDI of 0.22, and good stability for >6 months) is another drug of choice, with an increased CNS drug concentration and half-life when studied by Esposito et al. for the treatment of PD [200]. Apomorphine and ropinirole are other dopaminergic agonists, given through oral and intranasal routes, respectively, which have shown encouraging results in vitro, ex vivo and in vivo PD rat models [202]. SLNs loaded with levodopa, bromocriptine, rotigotine, apomorphine, and ropinirole are briefly detailed, alongside their functionality, in Table 4.

### 8.3. Drug Loaded SLNs for Huntington’s Disease

HD is an autosomal dominant disorder resulting from a mutation in the huntingtin (HTT) gene, with severe neurological disturbances and phenotypes such as dementia, depression, schizophrenia, abnormal body movements, chorea, athetosis, oculomotor apraxia, bipolar disorders, and sometimes suicidal tendencies [234]. There is no successful treatment for HD. Several drugs can reduce the symptoms of HD, such as: FDA-approved tetrabenazine (Xenazine); deutetrabenazine (Austedo), haloperidol, risperidone (Risperdal), olanzapine (Zyprexa), quetiapine (Seroquel), amantadine (Gocovri ER, Osmolex ER), levetiracetam (Keppra, Elepsia XR, Spritam), and clonazepam (Klonopin) [235,236]. The treatment strategy for HD is still unsuccessful, due to the unusual behavior of the BBB as an obstacle for drug-crossing and targeting. Advanced SLN drug carriers can deliver the drug candidates targeting HD, as they are able to cross the BBB and reach the target site of CNS, resulting in better therapeutic activity. Curcumin-loaded SLNs are one such candidate; they were tested in vivo for the treatment of HD, reducing the severity [203]. This specialized SLN has been shown to recover the neuronal loss due to mitochondrial dysfunctions and oxidative stress in the HD brain. They can also be helpful in increasing the reduced glutathione (GSH) levels and superoxide dismutases (SOD) activity. Bhatt et al. studied a rosmarinic-acid-loaded SLN carrier’s intranasal administration [204] for HD, looking at its neuroprotective qualities. The acted by reducing oxidative stress in HD in the in vivo animal models. In Table 4, the curcumin-loaded SLN and rosmarinic-acid-loaded SLN are presented, alongside their functionalities.

### 8.4. Drug Loaded SLNs for Multiple Sclerosis

Multiple sclerosis (MS) disables the CNS along with the brain and spinal cord, where the insulating covers of nerve cells in the brain and spinal cord are deactivated and damaged [237]. Therefore, the failure of signal transmission in the brain results in a range of devastating physical, mental, and psychiatric problems [238,239,240]. Certain FDA-approved drugs are available for clinical use such as: cladribine (Mavenclad), dimethyl fumarate (Tecfidera), diroximel fumarate (Vumerity), fingolimod (Gilenya), monomethyl fumarate (Bafiertam), ozanimod (Zeposia), siponimod (Mayzent), teriflunomide (Aubagio), interferon beta-1a (Avonex, Rebif), interferon beta-1b (Betaseron, Extavia), glatiramer acetate (Copaxone, Glatopa), peginterferon beta-1a (Plegridy), alemtuzumab (Lemtrada), mitoxantrone hydrochloride, natalizumab (Tysabri), and ocrelizumab (Ocrevus) to manage the severity of the MS symptoms and conditions. The bioavailability and plasma drug concentration are unsatisfactory, which many drugs showing reduced pharmacological activity. The current SLN-based drug delivery strategy has been shown to improve the efficacy of some drugs in the treatment of MS. The in vivo study based on riluzole-loaded SLNs, synthesized by microemulsion (average diameter 88 ± 4; PDI 0.27 ± 0.03 nm), has shown better brain delivery of the drug through the BBB. In comparison to other organs of the body, the brain showed a higher accumulation of the drug enhancing its neuroprotective abilities during the progression of MS and ALS in a rat model [241]. The functionality of SLNs loaded with the peculiar drug riluzole-loaded SLN are briefly described (Table 4).

### 8.5. Drug Loaded SLNs for Brain Tumor and Cancer

There are various grades of brain tumor in both nonmalignant and malignant forms; among these, glioblastoma is most prevalent, with a high risk of recurrence without successful treatment modality. The main obstacle to this is the difficulty in the effective transport of anti-cancer drugs across the BBB, resulting in lower therapeutic efficacy [242,243]. An advanced nano-drug carrier system is a novel approach to delivering a specific anticancer drug in a target-specific manner without affecting the normal, healthy cells [244]. A broad range of drugs and their modifications have been investigated for their ability to treat glioblastoma, such as the SLNs of etoposide [205] and paclitaxel [206]. In vitro studies demonstrated that these had an enhanced inhibitory effect on the proliferation of glioma cell lines, which was performed more efficiently than when using the free drug alone. Another study was based on anti-EGFR receptor functionalized cationic solid lipid nanoparticles (CASLNs) synthesized by the microemulsion method. The study demonstrated that the specialized SLN has anti-proliferative activity in targeting malignant glioblastoma cells [245]. The follow-up studies included in vitro studies of SLN composites of various drugs, including doxorubicin, and etoposide along with various targeting groups including aprotinin, anti-melanotransferrin, folic acid, p-aminophenyl-α-d-manno-pyranoside, serotonergic 1B receptor subtype antagonist, 83-14 monoclonal antibody (8314Mab), anti-endothelial growth factor receptor, tamoxifen, and lactoferrin on HBMEC, human U87 malignant glioma, human astrocytes cell lines. The results showed SLNs to be nontoxic with anti-proliferative effects. This could be attributed to the ability of these drug-loaded SLNs to infiltrate the BBB, suggesting their potential therapeutic use in future clinics for the treatment of multiple forms glioblastoma [245,246,247,248,249]. Other studies were conducted on SLNs loaded with the anticancer drug edelfosine synthesized by ultrasonication homogenization, when tested in vitro in a C6 glioma cell line and in vivo in a C6 glioma xenograft tumor. The results showed an anti-proliferative effect, with higher accumulation in the brain tissue, and a significant reduction in tumor growth [250]. Further research on SLNs of cetyl palmitate, stabilized with Tween^®^ 60 or Tween^®^ 80, carried out internalization studies in vitro and in vivo, and the results showed that the nanoparticles were internalized, leading to the increased therapeutic efficacy of the drug in crossing the BBB [251]. Another study aiming to enhance permeation through an in vitro BBB model, looked at the SLNs loaded with resveratrol functionalized with a targeting moiety, proving their inherent ability to passively target the brain [252]. SLNs loaded with paclitaxel and bevacizumab synthesized by the fatty acid coacervation technique, were found to penetrate the BBB (hCMEC/D3 cell monolayer in vitro BBB model) [253]. Anticancer drugs such as etoposide, paclitaxel, camptothecin, and doxorubicin loaded in SLNs are briefly presented, along with their functionalities, in Table 4.

### 8.6. Drug Loaded SLNs for Epilepsy

Overactivation of the electrical conductivity of the brain results in epilepsy, a CNS disorder resulting in partial or generalized seizures [254]. Here, the limitations of the therapeutics also include the inadequate concentration of drug delivery at the target site of the brain, due to the BBB serving as an obstacle. Among the conventional and recently developed drug delivery strategies, a nano-technological SLN based approach has shown possible advancements in overcoming the existing limitations in the treatment of epilepsy [255]. Recent research findings have shown promising results for SLNs loaded with carbamazepine with better anticonvulsant effect than nanoemulged-loaded carbamazepine [209]. Similarly, muscimol SLNs [256] and amiloride loaded SLNs [257] were evidenced to have anticonvulsant effects, suppressing focal seizures in in vivo rat models with a better and more sustained release in comparison to the administration of free drugs only. Details of SLNs loaded with carbamazepine, diazepam, clonazepam, and raloxifene are briefly explained in Table 4.

### 8.7. Drug Loaded SLNs in Ischemic Stroke

Ischemic stroke is the abrupt loss of the neurological function of the brain, leading to permanent disability caused by the sudden loss of blood and oxygen supply [258]. There are several types of ischemic strokes, such as lacunar, cardioembolic, and cryptogenic strokes, and hemorrhagic strokes [259]. Although this deformity of the brain results in a maximum rate of morbidity and mortality in the worldwide population, there is no effective therapeutic solution to date. Furthermore, during an ischemic stroke the brain tissue damage is a progressive process. Ischemic stroke starts with hypoxia following a secondary consequence such as severe inflammation in the brain tissue and reactive oxygen species (ROS) production, and glutamate excitotoxicity. Gradually, brain edema, BBB damage, and nerve tissue damage result in associated disorders including neuronal cell death [260,261,262]. The major treatment approach should be based on how to reduce proinflammatory consequences and providing neuroprotection [263]. The current treatment strategies are futile, due to the restricted bioavailability of the drugs across the BBB. An advanced nanodrug delivery approach may contribute a novel treatment strategy to overcome the major hurdles during drug targeting for stroke management [264]. SLN carrier-based drug delivery is one of the current nanotechnological approaches looking at potential drug formulations for ischemic stroke therapeutics. Some primary study findings revealed that SLNs loaded with vincristine and temozolomide, synthesized by the high-shear homogenization technique, have a profound, sustained release, suggesting future clinical use as a controlled delivery system [265]. The SLNs loaded with curcumin (as an antioxidant) have also gained particular interest in terms of their use in stroke treatment [266]. This study reports that SLN-encapsulated curcumin has a better therapeutic effect compared to free curcumin in inhibiting acetylcholinesterase levels and enhancing glutathione (GSH), superoxide dismutase (SOD), and catalase level. Another study focused on SLNs containing baicalin, and their actions against ischemic stroke, revealing the neuroprotective properties of encapsulated baicalin with improved bioavailability and stability [267]. Vinpocetine loaded in a specialized SLN formulation may overcome the short-comings, such as a lower bioavailability and short half-life associated with free vinpocetine in the treatment of chronic cerebral vascular ischemia [215]. Another study developed a surface modified (with apolipoprotein E: ApoE) resveratrol-loaded SLN on its surface, which can be identified by low-density lipoprotein (LDL) receptors in the BBB. Hence, this functionalized SLN drug carrier showed better BBB permeability in in vitro cell model [268]. In ischemic rat models, neurobehavioral deficits were improved significantly by ferulic acid (FA) loaded NLC, with improved bioavailability and reduced oxidative stress and neurotoxicity [269]. In Table 4, SLNs of curcumin, daidzein, and vinpocetine are presented, along with their functionalities.

### 8.8. Drug Loaded SLN for Other Neurodegenerative Diseases

Oxidative stress is a general hallmark of major neurodegenerative disorders, leading to neuronal cell dysfunction and progressive death [270]. Glutathione (GSH), lipoic acid (LA), carnosine, and caffeic acid provide potent antioxidant assistance in counteracting the free radicals produced by the ROS [222,271]. One study revealed that SLNs encapsulating LA can be used for the topical delivery of LA as an antiaging agent [272], with enhanced stability and hydrophilicity [273]. Another study found that lipoyl-memantine (LA-MEM codrug)-loaded SLNs are an innovative approach, which are stable in simulated gastric and intestinal fluids, improving stability, solubility and absorption through the gastrointestinal tract. This suggests that they can cross the BBB at maximum concentrations. Furthermore, LA and MEM were released as the end product of hydrolysis, exhibiting therapeutic efficacy in a safe and nontoxic manner [273]. Idebenone is another antioxidant drug of choice, loaded into SLN as a potent drug delivery system to the brain [152,228]. The in vitro study on the primary cultures of rat cerebral cortex astrocytes showed that idebenone-loaded SLNs were able to inhibit 2,2′-azobis-(2-amidinopropane) dihydrochloride (APPH)-induced lactic dehydrogenase (LDH) release, and ROS production. This idebenone-loaded SLN could be an interesting carrier system to pass the BBB, enhancing drug bioavailability in the brain. Luteolin (LU, 5,7,30,40-tetrahydroxyflavone) -loaded SLNs, synthesized by hot-microemulsion [217], have been shown to reduce oxidative stress in vivo in the management of neurodegenerative disorders. Interestingly, in vitro studies showed that AD and PD associated with severe neurodegeneration could be treated by resveratrol (a natural polyphenolic flavonoid) and grape-extract-loaded solid lipid nanoparticles, which enhance the regeneration of damaged neurons through crossing the BBB [274,275]. SLNs encapsulating curcuminoids, idebenone, and luteolin SLN are briefly summarized in Table 4.

This review paper emphasized a large number of drugs loaded SLNs and their therapeutic evaluation having potential for the treatment of various neurological disorders in future clinics. Even more interestingly, the SLNs formulations have exhibited the potency of crossing the BBB in the in vitro and in vivo models. Nonetheless, few of them have been approved in clinical context only in the treatment of cardiac diseases and some cancers, and not for neurological disorders in crossing the BBB [276,277]. There is no relevant clinical study for SLN containing drug for neurological disorders. However, the clinical study showed that [278], in comparison to only melatonin, administration of SLN loaded with melatonin was adequate to obtain better pharmacological levels even in the early phase of critical illness, with a favorable pharmacokinetic profile. This could be useful to achieve a sleep-inducing effect. Encapsulation into lipid nano vectors might offer some advantages from a pharmacokinetics point of view. Transdermal administration may represent an effective alternative to mimic the endogenous pattern of melatonin blood levels, possibly helping in restoring the circadian cycle in critically ill patients. More substantially, the major unsatisfactory consequences discovered during preclinical study are an inconsistent result of the SLN loaded drug, showing variable pharmacokinetic profiles in different animal models during crossing the BBB. This deviation in results of drug efficacy may be due to diverse brain microenvironments in the animal models. In this scenario further critical research is prerequisite to evaluate the bioavailability of the SLNs in predicting their pharmacological action and drug uptake in animal models, whose neurophysiology closely resembles that of humans. Hence, there is a better possibility of clinical approval of the SLNs in treatment of neurological disorders in targeting the BBB and their further commercialization.

## 9. Conclusions

To date, the treatment of CNS disorders is an onerous task in the field of medicine. The rate of mortality and morbidity is still an unresolved issue associated with complex neuro-pathologies, as well as the mechanisms behind the disorders, and BBB serves as a barrier for most of the therapeutic drugs. Recent biomedical research has made considerable progress in understanding the BBB as a potential target for brain drug delivery. In this context, much attention should be paid to the BBB not only as physical barrier, but also as a novel therapeutic target for a specific kind of drug delivery to CNS for the treatment of brain disorders. Among the lipid-based advanced nano-drug-delivery carrier systems, SLNs and their modification as a new therapeutics method, aimed to overcome the hindrances caused by the BBB. They have shown improved pharmacological applications. Furthermore, due to their unique physicochemical nature they can deliver the active drug contents in a target-specific and controlled manner, with fewer possible toxicity issues. Moreover, SLNs offer clinical advantages for effective brain drug delivery, with reduced side effects, an increased drug half-life, and the possibility of enhancing drugs’ ability to cross the BBB. Despite this, SLN has certain limitations, such as a lower drug payload, the complex physical state of the lipid content, and stability problems during storage and administration (gelation, increase in particle size, drug expulsion). Based on the current drawbacks associated with SLNs, futuristic development is needed to make them an ideal CNS drug delivery approach for treating the maximum number of neurological disorders. Finally, although current SLN strategies could not successfully cure neurological disorders, technological advancements and better understanding of the BBB transport mechanism can provide new hope in the development of this novel therapeutic strategy. Besides this, the standardization of the modified synthetic strategies, optimization of the sterilization process, scaling up of the manufacturing processes, and current stability issues are some of the challenges that need to be overcome before SLNs are approved for clinical use.

## Figures and Tables

**Table 1:** Advantages of SLNs over polymeric nanoparticles.

The lipid raw materials for SLNs are cheaper than the polymers (which also need regulatory approval for clinical use) [7]. So, it can be scaled up in a cost-effective manner due to cheaper synthetic approaches such as high pressure homogenization, etc. [51]. SLNs can avoid organic solvent as per requirement during small scale or large-scale synthesis. Hence, these formulations are less toxic and biomimetic, biocompatible and biodegradable with less or no toxicity [51,52]. SLNs escape RES bypassing liver and spleen filtration due to their unique physicochemical properties [53,54]. Furthermore, Lipids of SLNs are physiological and biodegradable, and hence have better biocompatibility. On the other hand, there is a chance polymeric nanoparticles accumulate in the liver, spleen etc. [55]. Encapsulated drugs are more stable within SLN in comparison to the polymeric nanoparticle, so prolonged release profile even for months to years. Comparing with polymeric nanoparticles, SLNs prohibit leakage and protect the active drug component from degradation (photochemical, oxidative, and chemical degradation) by immobilizing within and resulting a stable formulation [44,56]. SLNs can encapsulate both hydrophilic and lipophilic drugs [41,57,58]. The bioavailability of hydrophobic drugs is improved when they are in SLNs formulation in comparison to polymeric nanoparticles due to the physiological stability of the lipids [59]. When SLNs are conjugated with ligands, the drug targeting capability is improved [60,61]. Provide opportunities for targeted and controlled release of drug [60,62,63].

**Table 2:** Disadvantages of SLNs.

Poor drug loading due to limited space in the lipid matrix [64,65,66,67]. Drug interaction with lipid matrix is very common, sometimes results in failure of desired SLNs production. During storage of SLNs, there may be chances of drug expulsion following polymeric transition [68,69]. Relatively high-water content is not favorable condition for SLNs formulation from various points of view.

**Table 3:** Synthesis procedures for SLNs.

Technique	Precursor	Temp.	Particle Size Formed	Advantage	Disadvantage	Reference
**Coacervation method**	Soap micellar solution	25 °C	200–1000 nm	Drug can be dissolved directly in the micellar solution allow an advantageous drug loading within SLN for many drugs including poorly water-soluble drugs, high encapsulation yield, protection to the bioactive molecules	Nonuniform sized particle formation	[122,123,124,125]
**Cryogenic micronisation**	Supercritical fluid emulsion/Drug lipid matrix	70 °C	1–500 µm	Powdered SLNs are directly formed	Sometimes solvent associated toxicity	[126]
**Electrospray**	Supercritical fluid emulsion/Matrix solution	5–10 °C	1 µm	Monodisperse lipid-based nano-and microparticles formation	Variation in particle size	[127,128]
**Gas Anti-Solvent (GAS) process**	Hydrophobic materials	25–55 °C	200–2000 nm	Nanosized hydrophobic material formation	Solvent system incompatibility	[129]
**Hot homogenization**	Emulsion	5–10 °C	50–1000 nm	On a large scale for parenteral emulsion preparation in a nontoxic way	Not suitable for encapsulation of hydrophilic and thermosensitive drugs, metal contamination	[43,130,131]
**Cold homogenization**	Emulsion	0–4 °C	50–100 µm	Homogenous drug distribution in the lipid matrix	Larger particle sizes and a broader size distribution	[132,133]
**Melt dispersion technique**	Emulsion	90 °C	1–250 µm	Both hydrophilic and lipophilic drugs can be encapsulated either from O/W or multiple W/O/W emulsions	Sometimes bigger size microparticles are formed	[134,135]
**Membrane contactor technique**	Supercritical fluid emulsion/Lipid and the drug	−80 °C	100–200 nm	Easy operation, formation through a membrane unit	Bigger size particle formation, nonuniformity	[136,137]
**Microemulsion cooling technique**	Microemulsion	40–75 °C	50–300 nm	Simple, cost-effective, ingredients are potentially biocompatible, well-defined and uniform solid nanoparticle formation, very high entrapment efficiencies of drugs within SLN	Use of a large concentration of surfactant and co- surfactant necessary for stabilizing nano droplets, limited solubilizing capacity for high-melting substances.	[81,138,139,140]
**Microemulsion dilution technique**	Microemulsion	37–55 °C	50–800 nm	Thermodynamically optimized structures; direct (O/W), reversed (W/O) and multiple (W/O/W and O/W/O) emulsions and SLN can be formed	Bigger size particles formed	[141,142,143,144,145,146]
**Particles from Gas-saturated Solutions/Suspensions (PGSS)**	Gas saturated solution and suspension/Lipid and the drug	31 °C	0.2–20 µm	Formation of solid particles or droplets, CO_2_ solubility of the compound is not necessary,	Bigger size particle, complex operating system	[147,148,149]
**PIT method**	Emulsion	5–10 °C	30–100 nm	Nano W/O emulsion and SLN can be formed by phase inversion method	Sometimes not suitable for thermosensitive substances	[150,151,152]
**Rapid Expansion of Supercritical Solutions (RESS)/supercritical fluid nucleation (SFN)**	Drug and coating material	35–45 °C	200–2000 nm	Used for lipid coated microparticle formation	Low solubility of the compounds	[129,153]
**Solvent diffusion from emulsions**	Organic solvent emulsion	40–50 °C	100–2000 nm	Lipophilic and hydrophilic drugs can be encapsulated	Sometimes toxic	[154,155,156,157]
**Solvent evaporation from emulsions**	Organic solvent emulsion	25 °C	30–500 nm	Both hydrophilic and lipophilic drugs can be encapsulated in SLN	Not biocompatible	[154,155,156,157]
**Solvent injection**	Organic solvent emulsion/Lipid and the drug	25 °C	100–500 nm	Easy operation	Sometimes toxic according to the nature of the solvents used, Particle size non-uniformity	[158,159]
**Spray congealing**	Supercritical fluid emulsion/Lipid and the drug	5–10 °C	50–2000 µm	SLN-solid dispersion from poorly water-soluble drugs	Bigger size SLN, may not be suitable for thermosensitive drugs	[160,161]
**Spray-drying**	Supercritical fluid emulsion/Liquid feed (emulsion, liposome)	25 °C	0.3–10 µm	Ease of parameter control for desired SLN formulation	Drug degradation sometimes occurs, nonuniform shape particle formation	[162,163,164]
**Supercritical Fluid Extraction of Emulsions (SFEE).**	Supercritical fluid emulsion/Lipid and the drug	5–10 °C	20–90 nm	Lipid nanosuspensions formation, uniform sized particle	Solvent, temperature and pressure conditions affect the SLN particle properties	[165,166]
**High shear/ high speed/ ultrasound homogenization**		5–10 °C	50–1000 nm	Wide spread process, easy to handle	Metal contamination, bigger size particle formation	[120,167,168,169]

**Table 4:** Applications of SLNs in neurological disorders.

CNS Disorder	SLNs	Function	Study Performed	Reference
**Alzheimer’s** **Disease**	Nicotinamide loaded SLN functionalized with polysorbate 80 (S80), phosphatidylserine (PS) or phosphatidic acid (PA)	Histone deacetylase (HDAC) inhibitor	In vivo	[189]
		Superior nasal mucoadhesion and permeation, extended drug release, reducing oxidative stress, superior pharmacodynamic performance	Via nose-to-brain in goat ex vivo, in vivo, and preclinical study	[190]
	SLNs sesamol	Reduced acetylcholinesterase activity, attenuated oxidative-nitrergic stress and inflammatory cytokines	In vivo study	[191]
	Galantamine loaded SLN	Enhanced bioavailability and improved drug delivery	In vitro	[192]
	Quercetin-loaded SLN	Reverse neurodegeneration	In vivo	[193]
	Piperine loaded SLN	Overcome poor water solubility and BBB permeation	In vivo	[194]
	SLN carrying phosphatidic acid or cardiolipin	High affinity for the Aβ peptide	In vitro	[195]
	Ferulic acid loaded SLN	Overcome permeability issues and reduce oxidative stress in Aβ-treated cells	In vitro	[196]
	Epigallocatechin3-gallate	Improving oral bioavailability and preventing brain Aβ plaque formation	In vivo	[197]
	Curcumin loaded SLN	To completely reverse brain alterations induced by aluminum	In vivo	[198]
**Parkinson’s** **Disease**	Levodopa loaded SLN	Physical stability and entrapment efficiency enhanced	In vitro	[199]
	Bromocriptine loaded SLN	To stabilize plasma levels and increase CNS drug concentration and half-life	In vivo	[200]
	Rotigotine loaded SLN aerosol	Oral inhalation improvement	In vitro	[200]
	Apomorphine loaded SLN	Oral administration to increase bioavailability	In vitro	[201]
	Ropinirole loaded SLN	Intranasal formulations for alternative administration route	In vitro and ex vivo	[202]
**Huntington’s** **Disease**	Curcumin loaded SLN	Ameliorating mitochondrial dysfunctions	In vivo	[203]
	Rosmarinic acid loaded SLN	To enhance brain-targeting efficiency through intranasal administration and ameliorate behavioral dysfunctions associated with HD	In vivo	[204]
**Multiple Sclerosis**	Riluzole loaded SLN	A higher capability to carry the drug into the brain and a lower indiscriminate biodistribution	In vivo	[80]
**Tumor/Cancer**	SLNs of etoposide	Enhanced inhibitory effect on proliferation of glioma cell lines	In vitro	[205]
	SLNs of paclitaxel	Enhanced bioavailability with tumor targeting	In vitro	[206]
	SLN loaded with camptothecin	Improve the circulation time and brain accumulation	In vivo	[207]
	SLN loaded with doxorubicin		In vivo	[208]
**Epilepsy**	SLN loaded with carbamazepine	Anticonvulsant effect	In vitro	[209]
	SLN loaded with diazepam	Significant and prolonged release observed and good encapsulation efficiency	In vitro	[210]
	SLN loaded with clonazepam	Enhanced blood–brain barrier permeability	In vitro and In vivo	[211]
	SLN loaded with raloxifene	Increase in oral bioavailability and lymphatic absorption and good physical stability	In vivo	[212]
**Stroke**	SLN of curcumin	Alleviated behavioral, oxidative, and nitrosative stress; acetylcholinesterase; and mitochondrial enzyme complex, and physiological parameters	In vivo and ex vivo	[213]
	Daidzein SLN	Protective effect suffering from ischemia-reperfusion by increased cerebral blood flow, reduced cerebrovascular resistance and brain targeting	In vitro	[214]
	Vinpocetine SLN	Target chronic cerebral vascular ischemia or stroke by brain targeting and sustained release	In vitro	[215]
**Neurodegeneration**	SLN encapsulating curcuminoids	Therapeutically effective	In vivo and pre-clinical Studies	[216]
	Idebenone loaded SLN	Improving brain delivery and reducing cytotoxicity and oxidative stress in astrocytes from rat cerebral cortex	In vitro	[152]
	Luteolin SLN	Improve the bioavailability and pharmacokinetics of compound	In vitro and in vivo	[217]
