# Correction: Satapathy et al. Solid Lipid Nanoparticles (SLNs): An Advanced Drug Delivery System Targeting Brain Through BBB. *Pharmaceutics* 2021, *13*, 1183

**DOI:** 10.3390/pharmaceutics18091078

**Published:** 2026-08-27

**Authors:** Mantosh Kumar Satapathy, Ting-Lin Yen, Jing-Shiun Jan, Ruei-Dun Tang, Jia-Yi Wang, Rajeev Taliyan, Chih-Hao Yang

**Affiliations:** 1Department of Pharmacology, School of Medicine, College of Medicine, Taipei Medical University, No. 250, Wu Hsing St., Taipei 110, Taiwan; mantoshbiotech@gmail.com (M.K.S.); d119096015@tmu.edu.tw (T.-L.Y.); d119101004@tmu.edu.tw (J.-S.J.); tang0803@tmu.edu.tw (R.-D.T.); 2Department of Medical Research, Cathay General Hospital, Taipei 22174, Taiwan; 3Graduate Institute of Medical Sciences, College of Medicine, Taipei Medical University, No. 250, Wu Hsing St., Taipei 110, Taiwan; jywang2010@tmu.edu.tw; 4Department of Neurosurgery, Taipei Medical University Hospital, Taipei 110, Taiwan; 5Neuroscience Research Center, Taipei Medical University, Taipei 110, Taiwan; 6Department of Pharmacy, Neuropsychopharmacology Division, Birla Institute of Technology and Science, Pilani 333031, India; taliyanraja@gmail.com

## Text Correction

There was an error in the original publication [1]. In Table 4, the entry for “Stroke—SLN of curcumin” was incorrectly described as an in vitro study [1]. The “Study performed” section should be corrected to In vivo and ex vivo.

The corrected Table 4 entry should read:

**Table 4 pharmaceutics-18-01078-t004:** Applications of SLNs in neurological disorders.

CNS Disorder	SLNs	Function	Study Performed	Reference
**Alzheimer’s** **Disease**	Nicotinamide loaded SLN functionalized with polysorbate 80 (S80), phosphatidylserine (PS) or phosphatidic acid (PA)	Histone deacetylase (HDAC) inhibitor	In vivo	[189]
		Superior nasal mucoadhesion and permeation, extended drug release, reducing oxidative stress, superior pharmacodynamic performance	Via nose-to-brain in goat ex vivo, in vivo, and preclinical study	[190]
	SLNs sesamol	Reduced acetylcholinesterase activity, attenuated oxidative-nitrergic stress and inflammatory cytokines	In vivo study	[191]
	Galantamine loaded SLN	Enhanced bioavailability and improved drug delivery	In vitro	[192]
	Quercetin-loaded SLN	Reverse neurodegeneration	In vivo	[193]
	Piperine loaded SLN	Overcome poor water solubility and BBB permeation	In vivo	[194]
	SLN carrying phosphatidic acid or cardiolipin	High affinity for the Aβ peptide	In vitro	[195]
	Ferulic acid loaded SLN	Overcome permeability issues and reduce oxidative stress in Aβ-treated cells	In vitro	[196]
	Epigallocatechin3-gallate	Improving oral bioavailability and preventing brain Aβ plaque formation	In vivo	[197]
	Curcumin loaded SLN	To completely reverse brain alterations induced by aluminum	In vivo	[198]
**Parkinson’s** **Disease**	Levodopa loaded SLN	Physical stability and entrapment efficiency enhanced	In vitro	[199]
	Bromocriptine loaded SLN	To stabilize plasma levels and increase CNS drug concentration and half-life	In vivo	[200]
	Rotigotine loaded SLN aerosol	Oral inhalation improvement	In vitro	[200]
	Apomorphine loaded SLN	Oral administration to increase bioavailability	In vitro	[201]
	Ropinirole loaded SLN	Intranasal formulations for alternative administration route	In vitro and ex vivo	[202]
**Huntington’s** **Disease**	Curcumin loaded SLN	Ameliorating mitochondrial dysfunctions	In vivo	[203]
	Rosmarinic acid loaded SLN	To enhance brain-targeting efficiency through intranasal administration and ameliorate behavioral dysfunctions associated with HD	In vivo	[204]
**Multiple Sclerosis**	Riluzole loaded SLN	A higher capability to carry the drug into the brain and a lower indiscriminate biodistribution	In vivo	[80]
**Tumor/Cancer**	SLNs of etoposide	Enhanced inhibitory effect on proliferation of glioma cell lines	In vitro	[205]
	SLNs of paclitaxel	Enhanced bioavailability with tumor targeting	In vitro	[206]
	SLN loaded with camptothecin	Improve the circulation time and brain accumulation	In vivo	[207]
	SLN loaded with doxorubicin		In vivo	[208]
**Epilepsy**	SLN loaded with carbamazepine	Anticonvulsant effect	In vitro	[209]
	SLN loaded with diazepam	Significant and prolonged release observed and good encapsulation efficiency	In vitro	[210]
	SLN loaded with clonazepam	Enhanced blood–brain barrier permeability	In vitro and In vivo	[211]
	SLN loaded with raloxifene	Increase in oral bioavailability and lymphatic absorption and good physical stability	In vivo	[212]
**Stroke**	SLN of curcumin	Alleviated behavioral, oxidative, and nitrosative stress; acetylcholinesterase; and mitochondrial enzyme complex, and physiological parameters	In vivo and ex vivo	[213]
	Daidzein SLN	Protective effect suffering from ischemia-reperfusion by increased cerebral blood flow, reduced cerebrovascular resistance and brain targeting	In vitro	[214]
	Vinpocetine SLN	Target chronic cerebral vascular ischemia or stroke by brain targeting and sustained release	In vitro	[215]
**Neurodegeneration**	SLN encapsulating curcuminoids	Therapeutically effective	In vivo and pre-clinical Studies	[216]
	Idebenone loaded SLN	Improving brain delivery and reducing cytotoxicity and oxidative stress in astrocytes from rat cerebral cortex	In vitro	[152]
	Luteolin SLN	Improve the bioavailability and pharmacokinetics of compound	In vitro and in vivo	[217]

## References

**Previously the citation was:** 213. Vickers, N.J. Animal communication: When I’m calling you, will you answer too? *Curr. Biol.*
**2017**, *27*, R713–R715.

**Now the correct reference is:** 213. Yallapu, M.M.; Nagesh, P.K.B.; Jaggi, M.; Chauhan, S.C. Therapeutic applications of curcumin nanoformulations. *AAPS J.*
**2015**, *17*, 1341–1356. https://doi.org/10.1208/s12248-015-9811-z.

The authors state that the scientific conclusions are unaffected. This correction was approved by the Academic Editor. The original publication has also been updated.
